# Triterpene Glycosides from the Sea Cucumber *Ocnus glacialis* Display Cytotoxic and Colony-Inhibiting Activity Against Cancer Cells

**DOI:** 10.3390/md24080269

**Published:** 2026-08-03

**Authors:** Alexandra S. Silchenko, Ekaterina A. Chingizova, Ekaterina S. Menchinskaya, Kseniya M. Tabakmakher, Anatoly I. Kalinovsky, Sergey A. Avilov, Roman S. Popov, Pavel S. Dmitrenok, Vladimir I. Kalinin

**Affiliations:** G.B. Elyakov Pacific Institute of Bioorganic Chemistry, Far Eastern Branch of the Russian Academy of Sciences, Pr. 100-Letya Vladivostoka 159, 690022 Vladivostok, Russia; chingizova_ea@piboc.dvo.ru (E.A.C.); ekaterinamenchinskaya@gmail.com (E.S.M.); tabakmakher_km@piboc.dvo.ru (K.M.T.); kaaniv@piboc.dvo.ru (A.I.K.); avilov_sa@piboc.dvo.ru (S.A.A.); popov_rs@piboc.dvo.ru (R.S.P.); paveldmt@piboc.dvo.ru (P.S.D.); kalininv@piboc.dvo.ru (V.I.K.)

**Keywords:** triterpene glycosides, sea cucumbers, *Ocnus glacialis*, glacialisoside, hemolytic activity, cancer cells, inhibition of colony formation and growth

## Abstract

As a result of investigation of glycosidic composition of the sea cucumber *Ocnus glacialis* (Cucumariidae, Dendrochirotida) two new glycosides, glacialisosides A (**1**) and B (**2**), were isolated. Their structures were established by in-depth analysis of ^1^H, ^13^C NMR, 1D TOCSY, and 2D NMR (^1^H,^1^H COSY, HMBC, HSQC, ROESY), in addition to HR-ESI mass spectra. The structures of the obtained desulfated derivatives **3**, **4** were elucidated by HR-ESI-MS and ESI-MS/MS. The aglycone moieties of these glycosides are known from other glycosides of four species belonging to the order Dendrochirotida. However, despite sharing common sugar compositions and architectures, the carbohydrate chains of **1**, **2** are novel due to the distinct positioning of sulfate groups. Glacialisosides A (**1**) and B (**2**) exhibit structural features shared with compounds from sea cucumbers of the orders Holothuriida, Elasipodida, and Dendrochirotida. The hemolytic and cytotoxic activities of compounds **1**–**4** were studied against human erythrocytes and four breast cancer cell lines (MCF-7, T-47D, MDA-MB-231, and MDA-MB-468), as well as the non-tumorigenic mammary epithelial cell line MCF-10A and the pancreatic epithelioid carcinoma cell line PANC-1. The sulfated native compounds **1** and **2** were significantly more potent than desulfated derivatives **3** and **4** across all tested cell lines, indicating a positive contribution of sulfate groups to bioactivity. Notably, the normal epithelial MCF-10A cells exhibited resistance to the membranolytic action of the glycosides, an important and favorable feature, particularly given the pronounced cytotoxicity observed against the triple-negative MDA-MB-231 cell line. Furthermore, glacialisoside A (**1**) demonstrated potent inhibitory activity against the formation and growth of MDA-MB-468 cell colonies, effectively blocking cell division even at concentrations below 0.2 μM and completely halting it at a dosage of 1 μM. Thus, the colony formation assay reveals a latent sensitivity of cancer cells—not only to the membranolytic action of triterpene glycosides, but also to the effects of these compounds relevant to other aspects of cell survival, division, and spread.

## 1. Introduction

Marine invertebrates belonging to the class Holothuroidea, commonly called sea cucumbers, are widely distributed in all the oceans, from shallow waters to significant depths. *Ocnus glacialis* is a species of sea cucumber primarily found in Arctic and northeast Atlantic waters that inhabits the high-latitude benthic environments and is a part of the macrofaunal epibenthic community. *O. glacialis* is a suspension feeder, capturing food particles from the water column using its branched tentacles, similarly to the other representatives of the order Dendrochirotida.

Sea cucumbers are marine invertebrates highly valued for their high protein, low fat nutritional profile and the potent bioactive compounds that underlie their use in traditional medicine. Their therapeutic potential is attributed to the presence of triterpene glycosides, chondroitin sulfates, proteins and polysaccharides [1,2,3,4,5,6,7,8,9,10,11,12,13,14,15].

Chemical investigation of sea cucumbers is promising for the discovery of new natural compounds with pharmacological potential—triterpene glycosides [16,17,18,19,20,21,22]. The study of sea cucumber glycosides leads to some interesting aspects worthy of investigation. Knowledge of their structures enables the analysis of structure–activity relationships (SAR). Furthermore, the broader the library of characterized compounds, the more accurate the predictions of activity for new derivatives based on quantitative SAR (QSAR) [23]. In addition, modern tools for chemical space assessment allow not only the search for potential molecular targets for the studied compounds but also the virtual optimization of the structures of the latter to facilitate chemical synthesis or enhance target activity.

Additionally, glycosides from holothurians possess the taxonomical specificity that allow these glycosides to be used as chemotaxonomic markers [24,25,26]. The analysis of the structural features of the glycosides from previously unstudied species or genera of sea cucumbers, combined with molecular-genetic approaches, can help clarify their systematics and phylogeny.

Despite the fact that the glycosidic compositions of a number of sea cucumber species have been studied, the majority of them remain unexplored. None of the representatives of the *Ocnus* genus have been chemically studied, including *Ocnus glacialis*. In continuation of our investigations of glycosides from sea cucumbers, we have isolated two new triterpene glycosides from *O. glacialis*, glacialisosides A (**1**) and B (**2**). Their structures were elucidated by extensive analyses of ^1^H, ^13^C NMR, 1D TOCSY, and 2D NMR (^1^H,^1^H COSY, HMBC, HSQC, and ROESY), and HR-ESI mass spectra. Additionally, a series of desulfated derivatives (DS) (glacialisoside A (**1**), DS-glacialisoside B (**3**), and di-DS-glacialisoside B (**4**)) was obtained from glacialisoside B (**2**) to assess their cytotoxic activity and to analyze structure–activity relationships. All original spectra are provided in Figures S1–S19 in the Supplementary Data. The hemolytic activity against human erythrocytes and the cytotoxicity against human breast cancer cell lines MCF-7 and T-47D, triple-negative MDA-MB-231 and MDA-MB-468, and the non-tumorigenic mammary epithelial cell line MCF-10A, as well as cytotoxicity against pancreatic carcinoma PANC-1 cells, were tested for the compounds **1**–**4**.

## 2. Results and Discussion

### 2.1. Structure Elucidation of Glycosides

**Figure 1:**
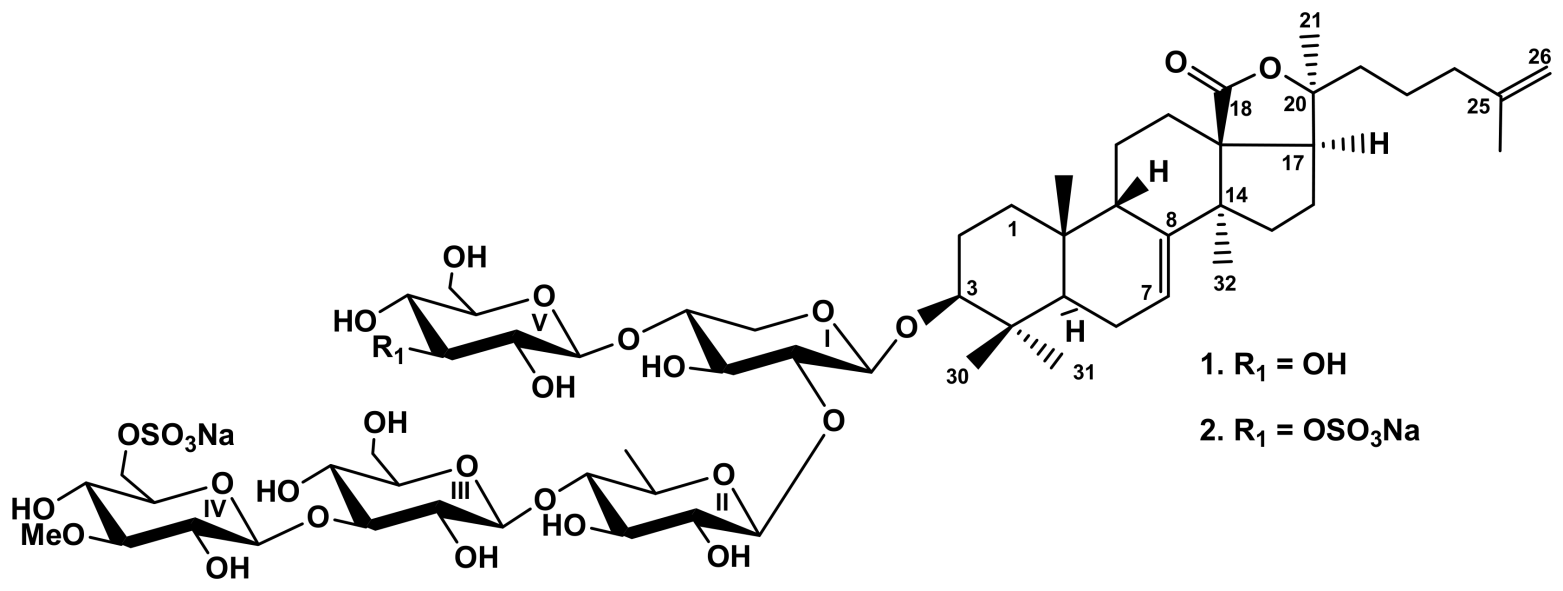
Chemical structures of the glycosides isolated from the sea cucumber *Ocnus glacialis*: **1**—glacialisoside A; **2**—glacialisoside B.

Specimens of *Ocnus glacialis* were collected during a scientific cruise of the research vessel “Akademik Oparin” in the Chukchi Sea near St. Gerald Island from a depth of 32 m, using a trawl. The crude glycosidic fraction (384 mg) was obtained from the concentrated ethanolic extract as a result of hydrophobic and silica gel column chromatography. After the repeated silica gel column chromatography, two fractions weighing ~30 and ~40 mg were obtained. Individual glycosides **1**, **2** (Figure 1) were isolated by subsequent HPLC on the reversed-phase column.

Since monosaccharide units in all known sea cucumber triterpene glycosides belong to the D-series, the sugar residues constituting the glycosides **1**, **2** were assigned the D configuration, based on biogenetic considerations. This determination is also confirmed by the frequently experimentally established D configuration in all monosaccharide residues of the glycosides of sea cucumbers, not only of the family Cucumariidae, to which *O. glacialis* belongs, but other families and orders also [27,28].

**Figure 2:**
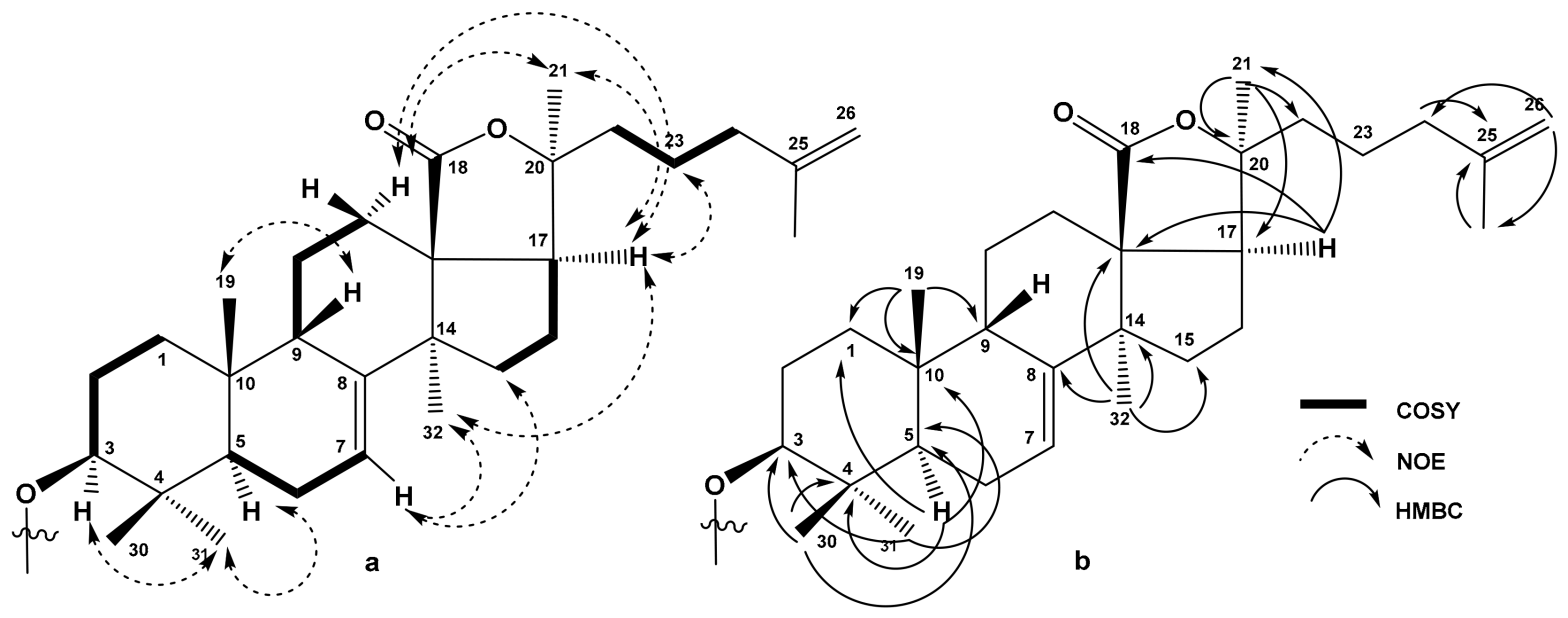
Key ^1^H,^1^H COSY, NOE- (**a**) and HMBC (**b**) correlations of the aglycone of glacialisosides A (**1**) and B (**2**).

An extensive analysis of the NMR spectra of the aglycones of **1** and **2** demonstrated the coincidence of the corresponding signals due to their identity with each other. Holostane-type aglycones, i.e., those having 18(20)-lactone in a lanostane backbone, are common for triterpene glycosides from sea cucumbers [29]. The presence of 18(20)-lactone was deduced from the characteristic signals of quaternary oxygen-bonded carbons at *δ*_C_ 180.8 (C-18) and 84.7 (C-20) (Table 1). The intranuclear double bond usually occupies the 7(8)- or 9(11)-position, resulting in the appearance of distinctive signals in ^13^C NMR spectra. The signals at *δ*_C_ 119.8 (C-7), 146.6 (C-8) indicated the positioning of double bond at C-7(8). Usually, the assigning of the signals of the aglycone part of glycosides starts from the finding of a C-3 signal, since this carbon is always observed at the low field region at δ_C_ ~88.0–89.0 due to glycosylation. Actually, the signal at δ_C_ 89.2 and corresponding proton signal at δ_H_ 3.19 (dd, *J* = 11.9; 3.5 Hz) deduced from the HSQC spectrum was assigned as a tertiary C-3 that was confirmed by NOE-correlation between H-3 and anomeric carbon of the first xylose residue (H-3/H-1 Xyl1). The protons H-1/H-2/H-3 formed an isolated spin system, as deduced from the ^1^H,^1^H COSY spectrum (Figure 2). The correlation H-3/H-5 in the ROESY spectrum allowed to proceed to the assignment of the signals of ring B of the polycyclic nucleus. The protons H-5/H-6/H-7 correlated in the ^1^H,^1^H COSY spectrum, and corresponding carbon signals were found by analysis of the HSQC spectrum. The signal of H-7 at *δ*_H_ 5.68 (m) and the presence of a long-range COSY correlation H-7/H-9 (5.68/3.36) due to π-electronic coupling confirmed the existence of a 7(8)-double bond. The further analysis of COSY correlations resulted in the finding of the signals of H-11 and H-12, which are parts of an isolated spin system, H-9/H-11/H-12, forming the ring C of the holostane backbone. The same pattern of structure elucidation was applied for the finding of the protons H-15, H-16, and H-17 (Figure 2). The signals of H-15 were deduced from the NOE-correlation H-7/H-15. The signals of quaternary carbons C-4, C-8, C-10, C-13, and C-14 were assigned from the HMBC correlations between the typical triterpenoid angular methyl groups CH_3_-19, -30, -31 and -32 and the corresponding carbons (Table 1, Figure 2). Additionally, the HMBC correlations H-17/C-18, H-21/C-20 and H-32/C-8 corroborated the presence of 18(20)-lactone and the position of the intranuclear double bond, correspondingly. The side chain structure was deduced step by step starting from the, common for glycosides, correlation of the H-21 methyl group with C-22, followed by COSY correlations H-22/H-23/H-24 and the assigning of corresponding carbons by HSQC spectrum. Two deshielded broad singlets, at δ_H_ 4.75 and 4.71 (H_2_-26), in the ^1^H NMR spectrum corresponded in HSQC to the carbon signal at δ_C_ 110.8 (C-26) that was distinctive for the terminal 25(26)-double bond. Actually, the HMBC correlations H-24/C-25 and H-27/C-25 showed the signal of quaternary vinylic carbon C-25 at δ_C_ 145.3 and confirmed the presence of the terminal double bond in the side chain. As the spatial structure of holostane-type aglycones is well-established, the orientation of methyls (CH_3_-19, CH_3_-21, CH_3_-30, CH_3_-31, and CH_3_-32) as well as the characteristic configuration of the C-9 chiral center were confirmed by NOE-correlations, namely, H-19/H-9, H-30; H-21/H-17; H-31/H-3, H-5; and H-32/H-7, observed in the spectra of **1** and **2** (Table 1, Figure 2, Table S1, Figures S1–S6 and S9–S15). The aglycone of **1** and **2** lacks any substituents in both the polycyclic nucleus and the side chain, and is identified as holosta-7,25-dien-3β-ol. This aglycone was found earlier in other glycosides of sea cucumbers of the order Dendrochirotida: *Cucumaria japonica* [27,29], *Colochirus robustus* [30], and genus *Psolus* [31].

The molecular formula of glacialisoside A (**1**) was determined to be C_60_H_95_O_29_SNa from the [M_Na_ − Na]^−^ ion peak at *m*/*z* 1311.5722 (calc. for C_60_H_95_O_29_S as 1311.5685) and [M_Na_ + Na]^+^ ion peak at *m*/*z* 1357.5467 (calc. for C_60_H_95_O_29_SNa_2_ as 1357.5470) in (−) and (+)HR-ESI-MS (Figure S8).

Analysis of the ^1^H, ^13^C NMR, and HSQC spectra of the carbohydrate moiety of glacialisoside A (**1**) (Table 2; Figures S1–S7) revealed the presence of five doublets of the anomeric protons, at *δ*_H_ 4.67 (d, *J* = 7.3 Hz), 4.97 (d, *J* = 7.7 Hz), 4.86 (d, *J* = 7.5 Hz), 5.12 (d, *J* = 8.1 Hz) and 4.88 (d, *J* = 7.7 Hz), and the signals of the corresponding anomeric carbons at *δ*_C_ 102.7–104.8. The anomeric proton chemical shifts and coupling constants values indicate *β*-glycosidic bonding of sugar units in the pentasaccharide chain [32]. The elucidation of the carbohydrate chain structure of **1** started with the identification of the NOE-correlation of H-3 of the aglycone (*δ*_H_ 3.19 (H-3, dd, *J* = 11.8; 3.5 Hz) with the anomeric proton of the first monosaccharide residue—xylose (Xyl1) (*δ*_H_ 4.67 (H-1 Xyl1, d, *J* = 7.3 Hz); this allowed all other signals of this residue to be deduced by subsequent analysis of ^1^H,^1^H COSY, 1D TOCSY, HSQC, and ROESY spectra. The deshielding of signals of C-2 and C-4 of Xyl1 to *δ*_C_ 82.7 and 77.4, respectively, due to glycosylation shifts indicated the attachment of the other sugar residues (quinovose—Qui2 and glucose—Glc5). Additionally, the coupling patterns of protons and NOE correlations H-1/H-3, H-1/H-5, and H-3/H-5 of the xylose, glucose and 3-O-methylglucose, as well as H-2/H-4 of quinovose (Table 2), unambiguously indicate the nature and conformation of each monosaccharide unit. The further analysis of the ROESY spectrum revealed the correlations H-2 Xyl1/H-1 Qui2 and H-4 Xyl1/H-1 Glc5. The following analysis of ^1^H,^1^H COSY, 1D TOCSY, HSQC, and ROESY spectra led to assignment of the signals of Qui2, in which H-4 (*δ*_H_ 3.55 (t, *J* = 8.5 Hz) was linked with the anomeric proton of glucose residue (H-1 Glc3, *δ*_H_ 4.86, d, *J* = 7.5 Hz) by *β*-glycosidic bond. The deshielding of C-4 Qui2 (*δ*_C_ 86.4) as well as HMBC correlation H-4 Qui2/C: 1 Glc3 and vice versa confirmed the position of the next monosaccharide residue. Using the same pattern of the analysis of NMR spectra, the structure of the oligosaccharide moiety of **1**, consisting of xylose (Xyl1) (as the first unit), quinovose (Qui2) (as the second unit), two glucoses (Glc3 and Glc5) (as the third and fifth residues) and 3-*O*-methylglucose (MeGlc4) as the terminal (fourth) unit, was established. HMBC correlations in addition to NOE-correlations corroborated the positions of glycosidic bonds (Table 2). The position of sulfate group, the presence of which was detected form the mass spectrum of **1**, was established based on *α*- and *β*-shifting effects, as observed from the signals of C-6 MeGlc4 (*δ*_C_ 67.1) and C-5 MeGlc4 (*δ*_C_ 75.6), correspondingly [32]. Thus, it was attached to C-6 of the terminal 3-*O*-methylglucose, and the carbohydrate chain of glacialisoside A (**1**) turned out to be new due to the combination of the branchpoint at C-4 Xyl1 and the presence and position of a sulfate group.

The (*+*)HR-ESI-MS/MS of **1** (Figure S8) demonstrated the fragmentation of the [M_Na_ + Na]^+^ ion at *m*/*z* 1357.5 leading to the appearance of fragment ions at *m*/*z* 1255.6 [M_Na_ + Na − NaSO_3_ + H]^+^, 1237.6 [M_Na_ + Na − NaHSO_4_]^+^, 1079.5 [M_Na_ + Na − MeGlcSO_3_Na + H]^+^, 921.2 [M_Na_ + Na − Agl + H]^+^, 759.2 [M_Na_ + Na − Agl − Glc + H]^+^, 609.1 [M_Na_ + Na − MeGlcSO_3_Na − Glc − Qui − Glc + H]^+^, 643.2 [M_Na_ + Na − Agl − MeGlcSO_3_Na + H]^+^, and 481.1 [M_Na_ + Na − Agl − MeGlcSO_3_Na − Glc + H]^+^, which confirmed the structure of sugar chain and the aglycone of glacialisoside A (**1**).

These data indicate that glacialisoside A (**1**) is 3*β-O*-{6-*O*-sodium sulfate-3-*O*-methyl-*β*-D-glucopyranosyl-(1→3)-*β*-D-glucopyranosyl-(1→4)-*β*-D-quinovopyranosyl-(1→2)-[*β*-D-glucopyranosyl-(1→4)]-*β*-D-xylopyranosyl}-holosta-7(8),25-diene.

The molecular formula of glacialisoside B (**2**) was determined to be C_60_H_94_O_32_S_2_Na_2_ from the [M_2Na_ − Na]^−^ ion peak at *m*/*z* 1413.5099 (calc. for C_60_H_94_O_32_S_2_Na as 1413.5073), [M_2Na_ − 2Na]^2−^ ion peak at *m*/*z* 695.2600 (calc. for C_60_H_94_O_32_S_2_ as 695.2590) and [M_2Na_ + Na]^+^ ion peak at *m*/*z* 1459.4862 (calc. C_60_H_94_O_32_S_2_Na_3_ as 1459.4857) in (−) and (+)HR-ESI-MS (Figure S16).

The carbohydrate chain of glacialisoside B (**2**) (Table 3; Figures S9–S15) consisted of five monosaccharide residues linked through *β*-glycosidic bonds; this was deduced from the analysis of ^1^H, ^13^C NMR, and HSQC spectra demonstrating the presence of five doublets of the anomeric protons, at *δ*_H_ 4.68 (d, *J* = 7.5 Hz), 4.99 (d, *J* = 8.4 Hz), 4.86 (d, *J* = 8.0 Hz), 5.12 (d, *J* = 7.9 Hz) and 4.91 (d, *J* = 7.7 Hz), and the signals of the corresponding anomeric carbons at *δ*_C_ 102.4–104.8. The signals of four monosaccharide residues composing the linear part of carbohydrate chain (from the first xylose (XylI) to terminal 3-*O*-methylglucose (MeGlcIV)) were very close in the ^13^C NMR spectra of **1** and **2**, indicating the same sugar composition and glycosidic bond positions in these glycosides. The signal of C-4 Xyl1 in the ^13^C NMR spectrum of **2** was deshielded to *δ*_C_ 77.7 due to its glycosylation with the fifth (glucose, Glc5) residue, similarly to **1**. NOE- and HMBC correlations H-4 Xyl1/H-1 (C-5) Glc5 and vice versa confirmed this. Additionally, the signals of C-3 (*δ*_C_ 84.4) and C-2 (*δ*_C_ 72.9) of Glc5 in the ^13^C NMR spectrum of **2** were deshielded and shielded, correspondingly, as compared to these signals in **1** (*δ*_C-3_ 77.3, *δ*_C-2_ 74.0). This was due to *α*- and *β*-shifting effects [32] of a sulfate group attached to C-3 Glc5 in **2**. This was in agreement with the MS data, indicating the presence of two sulfate groups in **2**. NOE- and HMBC correlations corroborated the positions of glycosidic bonds (Table 3).

The carbohydrate chain of glacialisoside B (**2**) has not been previously found among sea cucumber glycosides. It is characterized by an unusual structural feature—the attachment of a sulfate group to C-3 of glucose residue in the upper semi-chain (Glc5). Introduction of a substituent at C-3 of glucose residues occupying the third or fifth position in the carbohydrate chains prevents their further biosynthetic elongation by glycosyltransferases.

The (−)HR-ESI-MS/MS of glacialisoside B (**2**) (Figure S16) demonstrated the fragmentation of the [M_2Na_ − Na]^−^ ion at *m*/*z* 1413.5 that resulted in the appearance of fragment ions at *m*/*z* 1135.5 [M_2Na_ − Na − MeGlcSO_3_Na − H]^−^; 973.4 [M_2Na_ − Na − MeGlcSO_3_Na − Glc − H]^−^; and 827.4 [M_2Na_ − Na − MeGlcSO_3_Na − Glc − Qui − H]^−^, which corresponded to the sequential loss of monosaccharide residues. The (*+*)HR-ESI-MS/MS of **2** (Figure S16) demonstrated the fragmentation of the [M_2Na_ + Na]^+^ ion at *m*/*z* 1459.5 leading to the fragment ions at *m*/*z*: 1339.5 [M_2Na_ + Na − NaHSO_4_]^+^, 1023.1 [M_2Na_ + Na − Agl + H]^+^, 873.4 [M_Na_ + Na − MeGlcSO_3_Na − Glc − Qui + H]^+^, 759.2 [M_Na_ + Na − Agl − GlcSO_3_Na + H]^+^, 609.1 [M_Na_ + Na − MeGlcSO_3_Na − Glc − Qui − GlcSO_3_Na + H]^+^, and 481.1 [M_Na_ + Na − Agl − MeGlcSO_3_Na − GlcSO_3_Na + H]^+^, confirming the whole structure of **2**.

These data indicate that glacialisoside B (**2**) is 3*β-O*-{6-*O*-sodium sulfate-3-*O*-methyl-*β*-D-glucopyranosyl-(1→3)-*β*-D-glucopyranosyl-(1→4)-*β*-D-quinovopyranosyl-(1→2)-[3-*O*-sodium sulfate-*β*-D-glucopyranosyl-(1→4)]-*β*-D-xylopyranosyl}-holosta-7(8),25-diene.

**Figure 3:**
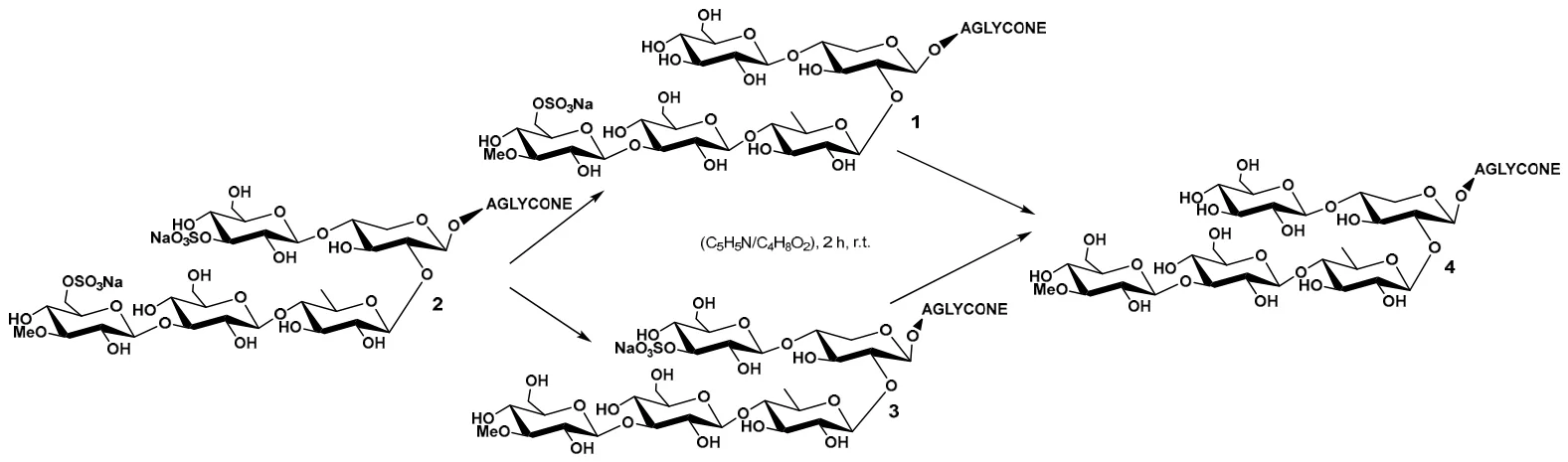
The products of solvolytic desulfation of glacialisoside B (**2**): **1**—glacialisoside A; **3**—mono-desulfated derivative DS-glacialisoside B; and **4**—di-desulfated derivative di-DS-glacialisoside B.

Glacialisoside B (**2**), bearing two sulfate groups, was subjected to solvolytic desulfation, affording three desulfated derivatives. Their membranolytic activity was studied; this was followed by structure–activity relationship (SAR) analysis to evaluate the influence of the number and positions of sulfate groups (Figure 3). A mixture of formed derivatives was separated by micro-column chromatography on silica gel using the stepped gradient of solvents CHCl_3_/EtOH/H_2_O in different ratios. The obtained compounds were: glacialisoside A (**1**), formed as a result of elimination of one sulfate group from C-3 Glc5, and bearing the sulfate group at C-6 MeGlc4; DS-glacialisoside B (**3**)—the glycoside lacking a sulfate at C-6 MeGlc4 but having it at C-3 Glc5; and di-DS-glacialisoside B (**4**))—a totally desulfated derivative of glacialisoside B (**2**) (Figure 3). The structures of these derivatives were established by HR-ESI-MS and HR-ESI-MS/MS data. The small amounts of the obtained substances did not allow us to acquire the NMR spectra.

The molecular formula of the product obtained as a result of the solvolytic desulfation, compound **1,** was determined to be C_60_H_95_O_29_SNa from the [M_Na_ − Na]^−^ ion peak at *m*/*z* 1311.5697 (calc. for C_60_H_95_O_29_S as 1311.5685) and [M_Na_ + Na]^+^ ion peak at *m*/*z* 1357.5460 (calc. for C_60_H_95_O_29_SNa_2_ as 1357.5470) in (−) and (+)HR-ESI-MS (Figure S17), and coincided with the molecular formula of native glacialisoside A (**1**). Its (*+*)HR-ESI-MS/MS o spectrum demonstrated the fragmentation of the [M_Na_ + Na]^+^ ion at *m*/*z* 1357.5 resulting in the fragment ion at *m*/*z* 1079.5 [M_Na_ + Na − MeGlcSO_3_Na + H]^+^, corresponding to the loss of sulfated 3-*O*-methylglucose residue, and indicating the Glc5 residue was desulfated. The fragment ion-peaks at *m*/*z* 1237.6 [M_Na_ + Na − NaHSO_4_]^+^, 921.2 [M_Na_ + Na − Agl + H]^+^, 759.2 [M_Na_ + Na − Agl − Glc + H]^+^, 609.1 [M_Na_ + Na − MeGlcSO_3_Na − Glc − Qui − Glc + H]^+^, and 481.1 [M_Na_ + Na − Agl − MeGlcSO_3_Na − Glc + H]^+^ were the same as in the MS/MS spectrum of native glacialisoside A (**1**), corroborating their identity. Thus, the structure of desulfated derivative **1** was deduced to be 3*β-O*-{6-*O*-sodium sulfate-3-*O*-methyl-*β*-D-glucopyranosyl-(1→3)-*β*-D-glucopyranosyl-(1→4)-*β*-D-quinovopyranosyl-(1→2)-[*β*-D-glucopyranosyl-(1→4)]-*β*-D-xylopyranosyl}-holosta-7(8),25-diene.

The molecular formula of DS-glacialisoside B (**3**) was the same as for **1** (C_60_H_95_O_29_SNa), as was deduced from the [M_Na_ – Na]^−^ ion peak at *m*/*z* 1311.5678 (calc. for C_60_H_95_O_29_S as 1311.5685) and [M_Na_ + Na]^+^ ion peak at *m*/*z* 1357.5458 (calc. for C_60_H_95_O_29_SNa_2_ as 1357.5470) in (−) and (+)HR-ESI-MS (Figure S18). The (*+*)HR-ESI-MS/MS spectrum of **3** demonstrated the fragment ion-peak at *m*/*z* 1093.5 [M_Na_ + Na − GlcSO_3_Na + H]^+^, corresponding to the loss of sulfated glucose residue and indicating that 3-O-methylglucose residue was desulfated. Actually, the ion peaks at *m*/*z* 657.2 [M_Na_ + Na − Agl − GlcSO_3_Na + H]^+^ and 507.2 [M_Na_ + Na − MeGlc − Glc − Qui − GlcSO_3_Na + H]^+^ confirmed this. Additionally, in the (−)HR-ESI-MS/MS spectrum of **3** the peaks of fragment ions were observed at *m*/*z* 1135.5 [M_Na_ − Na − MeGlc − H]^−^, 973.4 [M_Na_ − Na − MeGlc − Glc − H]^−^, and 827.3 [M_Na_ − Na − MeGlc − Glc − Qui − H]^−^, which is in good agreement with partial desulfation of **2** with the loss of a sulfate group from the terminal 3-*O*-methylglucose residue. Thus, DS-glacialisoside B (**3**) is 3*β-O*-{3-*O*-methyl-*β*-D-glucopyranosyl-(1→3)-*β*-D-glucopyranosyl-(1→4)-*β*-D-quinovopyranosyl-(1→2)-[3-*O*-sodium sulfate-*β*-D-glucopyranosyl-(1→4)]-*β*-D-xylopyranosyl}-holosta-7(8),25-diene.

The molecular formula of di-DS-glacialisoside B (**4**) was determined to be C_60_H_96_O_26_ from the [M − H]^−^ ion peak at *m*/*z* 1231.6117 (calc. for C_60_H_95_O_26_ as 1231.6117) and [M + Na]^+^ ion peak at *m*/*z* 1255.6076 (calc. for C_60_H_96_O_26_Na as 1255.6080) in (−) and (+)HR-ESI-MS (Figure S19), which corresponded to a totally desulfated derivative of **2** lacking sulfate groups. The (*+*)HR-ESI-MS/MS spectrum of di-DS-glacialisoside B (**4**) demonstrated the fragment ion-peaks at *m*/*z* 1079.5 [M + Na − MeGlc + H]^+^, 917.5 [M + Na − MeGlc − Glc + H]^+^, 819.3 [M + Na − Agl + H]^+^, 771.4 [M + Na − MeGlc − Glc − Qui + H]^+^, 657.2 [M + Na − Agl − Glc + H]^+^, and 481.1 [M + Na − Agl − MeGlc − Glc + H]^+^, corroborating the aglycone and oligosaccharide chain structure of **4.** The most intensive fragment ion-peaks in (−)HR-ESI-MS/MS spectrum of di-DS-glacialisoside B (**4**), at *m*/*z* 1055.5 [M − H − MeGlc + H]^−^, 893.5 [M − H − MeGlc − Glc + H]^−^, and 731.4 [M − H − MeGlc − Glc − Qui + H]^−^, also indicated the absence of sulfate groups in the chain of **4**. Thus, di-DS-glacialisoside B (**4**) is 3*β-O*-{3-*O*-methyl-*β*-D-glucopyranosyl-(1→3)-*β*-D-glucopyranosyl-(1→4)-*β*-D-quinovopyranosyl-(1→2)-[*β*-D-glucopyranosyl-(1→4)]-*β*-D-xylopyranosyl}-holosta-7(8),25-diene.

Compounds **1** and **2**, isolated from the sea cucumber *Ocnus glacialis*, contain an aglycone previously known from four holothurian species of the order Dendrochirotida [19,27,29,30,31]. The carbohydrate chains of glacialisosides A (**1**) and B (**2**) share a common sugar composition and architecture but differ from all previously known glycosides in the position of the sulfate groups. An oligosaccharide moiety similar in structure to that of **1** and **2**, but lacking sulfate groups, has been found in *Holothuria forskali* [33], *Bohadschia cousteaui* [34], and *Holothuria grisea* [35]—representatives of the order Holothuriida—as well as in *Kolga hyalina* [19], a member of the order Elasipodida. Sulfated glycosides with sugar chains structurally close to those of glacialisosides, yet differing in the positions of the sulfate groups, have been reported from another representative of the order Elasipodida, *Rhipidothuria racovitzai* [19]. Notably, the aglycones of these glycosides differ substantially from those of *O. glacialis*. Tri- and tetrasulfated glycosides possessing a carbohydrate chain of the same composition and architecture as in **1** and **2** have been isolated from *Psolus fabricii* [36]. In addition, a monosulfated trioside from *P. fabricii* shares the same aglycone as glacialisosides A (**1**) and B (**2**). Thus, the glycosides of *O. glacialis* exhibit common structural features with compounds isolated from sea cucumbers belonging to different orders due to parallel chemical evolution of glycosides in different taxa of Holothuriida, yet they also possess unique characteristics that establish them as new compounds. Additionally, the species *Ocnus glacialis* previously had the original name *Cucumaria glacialis*, while the structures of the glycosides showed the absence of characteristic features inherent to the glycosides of representatives of genus *Cucucmaria* [29]. That unambiguously confirms the validity of its assignment to another genus, *Ocnus glacialis*.

### 2.2. Biological Activity of Glacialisosides A (***1***) and B (***2***) and Desulfated Derivatives ***3***, ***4***

The cytotoxic activity of the compounds **1**–**4** was studied against human erythrocytes as well as on four types of human breast cancer cells (MCF-7 and T-47D, two triple negative (TNBC) lines; MDA-MB-231 (mesenchymal-like); and MDA-MB-468 (basal-like)), as well as the non-tumorigenic mammary epithelial cell line MCF-10A and pancreatic epithelioid carcinoma PANC-1 cell line. Cucumarioside A_0_-1 was used as the positive control since it demonstrated high cytotoxic activity against MDA-MB-231 and T-47D cells and was earlier selected as a lead compound for deep investigation of anticancer activity [37]. Cytotoxic activity against all the selected cell lines was assessed using the MTT method (Table 4).

Both native glycosides **1** and **2** exhibited strong hemolytic action against human erythrocytes due to non-specific binding with membrane sterols and lipids, leading to membrane disruption. As usual, the red blood cells were more sensitive to the membranolytic action of the glycosides, as compared to the cancer cells (Table 5), particularly due to the enrichment of red blood cells in cholesterol, with which the triterpene core of glycosides interacts [38,39]. The selectivity index of each studied compound relative to hemolysis was less than 1 for the cell lines tested (Table 5).

Regarding the dependence of the membranolytic action of tested compounds on the presence and position of sulfate groups, a clear tendency was observed: the sulfated native compounds are significantly more effective than the desulfated derivatives, against all tested cell lines (Table 4). The hemolytic action of monosulfated glacialisoside A (**1**), bearing a sulfate group at C-6 MeGlc4, is two-fold stronger than that of disulfated glacialisoside B (**2**), and seven-fold stronger than the effect of **3**, having one sulfate group at C-3 Glc5. Hence, the sulfation of C-6 MeGcl4 has a significant positive impact on the activity. In contrast, the sulfate group attached to an unusual position—C-3 of the terminal glucose residue (Glc5) in the upper semi-chain—exerts a slightly negative influence on the membranolytic action, compared with the more favorable and common position at C-6 of the terminal residue in the lower semi-chain. However, elimination of all sulfate groups led to a marked decrease in activity, with the fully desulfated derivative **4** being the least active compound against all tested cell lines. Thus, sulfation at C-3 of Glc5 retains a positive contribution relative to the complete absence of sulfate groups.

These observations are in full agreement with previous findings indicating the complex and ambiguous nature of structure–activity relationships (SAR) inherent to triterpene glycosides [40]. As previously demonstrated using quantitative structure–activity relationship (QSAR) methodology, this complexity arises from the cumulative effects of numerous minor contributions from individual structural descriptors, which collectively result in a significant overall impact [40]. Nevertheless, the contribution of a specific structural feature to activity can become evident when comparing glycosides with identical core structures with defined differences. Earlier studies showed that the presence of sulfate groups at C-6 of monosaccharide units enhances the membranotropic action of glycosides with pentasaccharide chains branched at C-4 of Xyl1, in contrast to the activity of pentasides branched at C-2 of Qui2. Meanwhile, sulfation at C-2 of hexose residues exerted a strongly negative influence on the membranotropic activity. Collectively, these data indicate that sulfate groups exert an ambiguous effect on glycoside activity, which is highly dependent on the architecture of the carbohydrate chain [40].

Importantly, the QSAR model revealed a positive contribution of the sulfate group at C-3 of the terminal residue in the lower semi-chain of hexaosides, cladolosides T and T_1_, isolated from the sea cucumber *Cladolabes schmeltzii* [40]. A similar effect was observed for chilensosides E and F, compounds isolated from *Paracaudina chilensis* [41], both bearing a sulfate group at C-3 of the third terminal glucose residue in the lower semi-chain. These compounds demonstrated activity comparable to that of glycosides lacking a sulfate group at this unusual position. Taken together, these findings support the conclusion that sulfation at C-3 of various monosaccharide residues located at diverse positions within the carbohydrate chain architecture has a neutral or slightly positive impact on the membranolytic activity of the glycosides.

Notably, with respect to different cancer cells, the action of tested glycosides is almost equal or, more precisely, **2** is slightly more cytotoxic than **1**, while both desulfated derivatives **3** and **4** are inactive. These differences are likely attributable to variations in the compositions of the cell membranes across the different cell lines.

The most pronounced cytotoxicity was observed against the TNBC cell line MDA-MB-231, while the other cancer cell types were relatively resistant to the action of the glycosides. Importantly, normal epithelial MCF-10A cells remained unaffected, underscoring the selective toxicity of the tested glycosides toward malignant cells. This favorable selectivity profile, combined with their potent activity against an aggressive and clinically challenging cancer subtype, positions these compounds as promising candidates for further pharmacological evaluation and development. Similarly to the majority of natural compounds, sea cucumber triterpene glycosides are obviously not single-target compounds, but act as pleiotropic agents through several interconnected pathways demonstrating distinct effects in relation to different cell lines or in diverse assays. The obtained data demonstrate that the prolonged action of the glycosides checked in the colony formation assay led to significant inhibition of clonogenic potential in MDA-MB-468 cells along with MDA-MB-231 cells, despite the low cytotoxic action of the tested compound against the former cell line.

**Figure 4:**
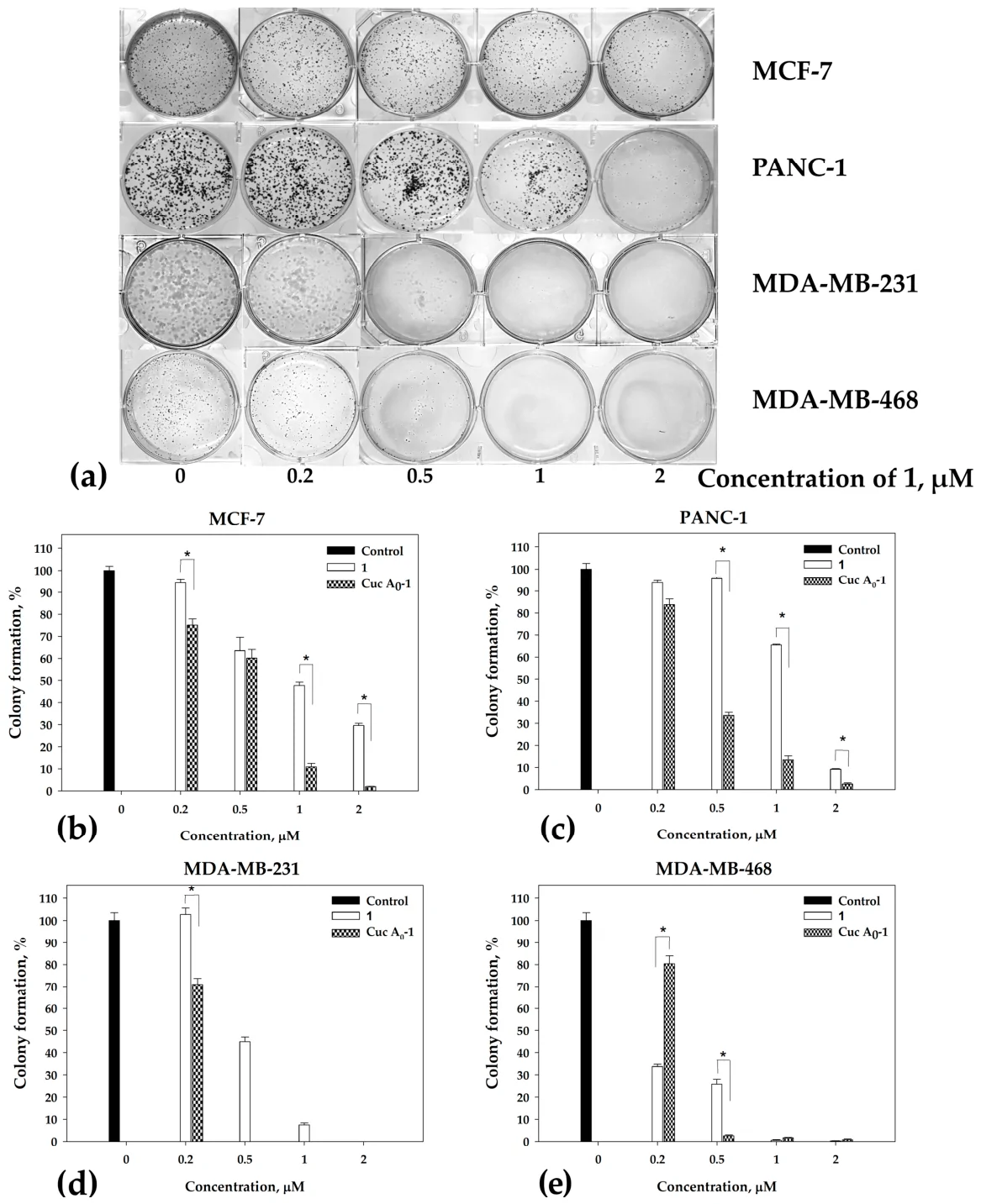
The inhibition of colony formation in MCF-7 (**a**,**b**), PANC-1 (**a**,**c**), MDA-MB-231 (**a**,**d**), and MDA-MB-468 (**a**,**e**) cells by glacialisoside A (**1**) and Cuc A_0_-1 (positive control), in different concentrations. Data are presented as means ± SEM. * *p* value < 0.05 considered significant.

The ability of an individual cell to maintain its reproductive function—specifically, to divide and grow into a detectable colony—is the fundamental principle behind the colony formation assay, a common method for assessing cell survival in vitro. This process serves as a direct correlation with in vivo pathophysiology, wherein the expansion of cancer cells leads directly to metastatic spread. The ability of the most active glycoside, glacialisoside A (**1**), to inhibit the formation and growth of colonies of MDA-MB-231, MDA-MB-468, MCF-7 and PANC-1 cancer cells was investigated using a range of non-toxic concentrations (Figure 4). A dose-dependent effect was observed against all tested cell lines, but it was most significant in relation to TNBC MDA-MB-231 and MDA-MB-468 lines (Table 6).

It is interesting to note that MDA-MB-468 cells characterized by notable epidermal growth factor receptor (EGFR) amplification were more sensitive to the colony-inhibiting action of **1** than were the cells MDA-MB-231, in contrast to the cytotoxic action of the glycoside, indirectly confirming the multi-target action of glycosides. At a concentration of 0.2 μM, compound **1** inhibited colony growth of MDA-MB-468 cells by 66.3%, while it did not affect the MDA-MB-231 cells. An increased concentration of **1** (0.5 μM) led to more pronounced inhibition of colony formation in both lines: 55.1% for MDA-MB-231 cells and 74.2% for MDA-MB-468 cells. A dosage of 1 μM completely blocked the clonogenic potential of MDA-MB-468 cells and inhibited that of MDA-MB-231 cells by 92.5%. A concentration of 2 μM halted colony formation in both the MDA-MB-231 and MDA-MB-468 lines and inhibited this property in MCF-7 cells by 70.4%, and in PANC-1 cells by 91% (Figure 4).

The results obtained show that the IC_50_ for colony formation is lower than this value for the cytotoxicity of **1** (Table 4 and Table 6). To improve clarity, we also calculated the selectivity index of colony formation inhibition relative to hemolytic activity. The data presented in Table 7 indicate that compound **1**, with a selectivity index greater than 1, effectively inhibits colony formation by the MDA-MB-468 cell line. The reference compound cucumarioside A_0_-1 inhibits colony formation across all tested cell lines with a selectivity index greater than 1.

The observed discrepancy between the cytotoxic and colony-inhibiting effects of glacialisoside A (**1**) can be explained by fundamental differences in the assays. While the MTT test measures short-term (24 h) metabolic activity and primarily detects acute cell death, the clonogenic assay assesses the long-term proliferative capacity of individual surviving cells over 10–14 days. The low (sub-cytotoxic) concentrations of the glycoside may cause cumulative or delayed damage—such as persistent membrane instability and disruption of cell adhesion—which does not immediately kill the cells but irreversibly blocks their ability to undergo sustained division. Furthermore, the higher sensitivity of MDA-MB-468 cells in the clonogenic assay, compared to MDA-MB-231 cells, correlates with their distinct molecular profiles, which may render the former more vulnerable to the long-term antiproliferative effects of the glycoside. Thus, the colony formation assay reveals a “latent” sensitivity of cancer cells—not only a sensitivity to the membranolytic action of triterpene glycosides, but also to their effects on other aspects of cell survival, division, and spread.

## 3. Materials and Methods

### 3.1. General Experimental Procedures

A PerkinElmer 343 Polarimeter (PerkinElmer, Waltham, MA, USA) was used for specific rotation measuring; NMR spectra were registered on an Avance III 700 Bruker FT-NMR spectrometer (Bruker BioSpin GmbH, Rheinstetten, Germany) (700.13/176.04 MHz (^1^H/^13^C, 30 °C, *δ*_C_ 148.9 resonance of C_5_D_5_N/D_2_O (4/1) for ^13^C and *δ*_H_ 7.21 resonance of C_5_D_5_N/D_2_O (4/1) for ^1^H used as the references, BBO probe); ESI MS (negative and positive ion modes) spectra were obtained on an Agilent 6510 Q-TOF apparatus (Agilent Technology, Santa Clara, CA, USA), sample concentration 0.01 mg/mL; HPLC was conducted on an Agilent 1260 Infinity II equipped with a differential refractometer (Agilent Technology, Santa Clara, CA, USA); the column used was a Phenomenex Synergi Fusion RP (10 × 250 mm, 5 μM) (flow rate of 1.5 mL/min) (Phenomenex, Torrance, CA, USA).

### 3.2. Animals and Cells

Specimens of the sea cucumber *Ocnus glacialis* (family Cucumariidae; order Dendrochirotida) were collected in September of 2016 during a scientific cruise of the research vessel “Akademik Oparin” in the Chukchi Sea near St. Gerald Island, from a depth of 32 m using a trawl. Sea cucumbers were identified taxonomically by S.S. Dautov; voucher specimens are preserved in the collection of the Museum of A.V. Zhirmunsky National Scientific Center of Marine Biology, Vladivostok, Russia.

Human erythrocytes were obtained from the Regional Blood Transfusion Station (Vladivostok, Russia). The American Type Culture Collection (ATCC; Manassas, VA, USA) provided the human cell lines used in this study: non-malignant mammary epithelial cells MCF-10A (CRL-10317) and breast adenocarcinoma cells T-47D (HTB-133), MCF-7 (HTB-22), MDA-MB-468 (HTB-132), and MDA-MB-231 (CRM-HTB-26), as well as pancreatic carcinoma cells PANC-1 (CRL-1469). Culture media, fetal bovine serum (FBS), and penicillin/streptomycin antibiotics were purchased from Biolot (St. Petersburg, Russia).

The MCF-10 A cells were cultured in DMEM/F12 medium with 20% FBS, 20 ng/mL EGF (Sci-Store, Moscow, Russia) and 1% penicillin/streptomycin. The T-47D cells were cultured in RPMI-1640 medium with 10% FBS and 1% penicillin/streptomycin. The MCF-7, MDA-MB-468 and MDA-MB-231 cells were cultured in MEM medium with 10% FBS and 1% penicillin/streptomycin. The PANC-1 cells were cultured in DMEM medium with 10% FBS and 1% penicillin/streptomycin.

All cell lines were incubated at 37 °C in an incubator with an atmosphere of 5% CO_2_.

### 3.3. Extraction and Isolation

Sea cucumbers (5 pieces) were minced and extracted twice with refluxing aqueous 65% EtOH. The dry weight of the residue was approximately 102 g. The combined extracts were concentrated to dryness in vacuo, dissolved in H_2_O, and chromatographed on a hydrophobic sorbent Polychrom-1 column (Biolar, Olaine, Latvia) to eliminate inorganic salts and impurities. As a result, ~1000 mg of glycosidic sum was obtained, which was subjected to silica gel column chromatography in stepped gradient with solvent systems CHCl_3_/EtOH/H_2_O (10:10:1.7 → 4:5:1) to give 384 mg of crude glycosidic fraction. After the repeated silica gel column chromatography in solvent system CHCl_3_/EtOH/H_2_O (10:10:1.7), two fractions, I and II, weighing 30 and 40 mg, were obtained. They were further separated by HPLC using a Phenomenex Synergi Fusion RP (Phenomenex, Torrance, CA, USA) column. HPLC of fraction I with CH_3_CN/H_2_O/NH_4_OAc (1M water solution) in a ratio of (50/48.5/1.5) as the mobile phase yielded one main compound—glacialisoside A (**1**) (7 mg)—and another subfraction (5 mg) composed of a mixture of glycosides and lipids. Fraction II (40 mg), subjected to HPLC with MeOH/H_2_O/NH_4_OAc (1M water solution) in a ratio of (70/28.5/1.5) as the mobile phase, gave glacialisoside B (**2**) (5 mg).

#### 3.3.1. Glacialisoside A (**1**)

Colorless powder; [α]_D_^20^−53° (*c* 0.1, H_2_O), mp 197 °C. Data of NMR: Table 1 and Table 2, Figures S1–S7. (−)HR-ESI-MS *m*/*z*: 1311.5722 (calc. 1311.5685) [M_Na_ − Na]^−^; (*+*)HR-ESI-MS *m*/*z*: 1357.5458 (calc. 1357.5470) [M_Na_ + Na]^+^; (+)ESI-MS/MS *m*/*z*: 1255.6 [M_Na_ + Na − NaSO_3_ + H]^+^, 1237.6 [M_Na_ + Na − NaHSO_4_]^+^, 1079.5 [M_Na_ + Na − MeGlcSO_3_Na (C_7_H_12_O_9_SNa) + H]^+^, 921.2 [M_Na_ + Na − Agl (C_30_H_45_O_2_) + H]^+^, 759.2 [M_Na_ + Na − Agl (C_30_H_45_O_2_) − Glc (C_6_H_10_O_5_) + H]^+^, 643.2 [M_Na_ + Na − Agl (C_30_H_45_O_2_) − MeGlcSO_3_Na (C_7_H_12_O_9_SNa) + H]^+^, 609.1 [M_Na_ + Na − MeGlcSO_3_Na (C_7_H_12_O_9_SNa) − Glc (C_6_H_10_O_5_) − Qui (C_6_H_10_O_4_) − Glc (C_6_H_10_O_5_) + H]^+^, 481.1 [M_Na_ + Na − Agl (C_30_H_45_O_2_) − MeGlcSO_3_Na (C_7_H_12_O_9_SNa) − Glc (C_6_H_10_O_5_) + H]^+^ (Figure S8).

#### 3.3.2. Glacialisoside B (**2**)

Colorless powder; [α]_D_^20^−64° (*c* 0.1, H_2_O), mp 222 °C. Data of NMR: Table 3 and Table S1, Figures S9–S15. (−)HR-ESI-MS *m*/*z*: 1413.5099 (calc. 1413.5073) [M_2Na_ − Na]^−^, 695.2600 (calc. 695.2590) [M_2Na_ − Na]^2−^; (*+*)HR-ESI-MS *m*/*z*: 1459.4862 (calc. 1459.4857) [M_2Na_ + Na]^+^; (−)ESI-MS/MS *m*/*z*: 1135.5 [M_2Na_ − Na − MeGlcSO_3_Na (C_7_H_12_O_8_SNa) − H]^−^, 973.4 [M_2Na_ − Na − MeGlcSO_3_Na (C_7_H_12_O_8_SNa) − Glc (C_6_H_10_O_5_) − H]^−^, 827.4 [M_2Na_ − Na − MeGlcSO_3_Na (C_7_H_12_O_8_SNa) − Glc (C_6_H_10_O_5_) − Qui (C_6_H_10_O_4_) − H]^−^; (+)ESI-MS/MS *m*/*z*: 1339.5 [M_2Na_ + Na − NaHSO_4_]^+^, 1023.1 [M_2Na_ + Na − Agl (C_30_H_45_O_2_) + H]^+^, 873.4 [M_2Na_ + Na − MeGlcSO_3_Na (C_7_H_12_O_8_SNa) − Glc (C_6_H_10_O_5_) − Qui (C_6_H_10_O_4_) + H]^+^, 759.2 [M_2Na_ + Na − Agl (C_30_H_45_O_2_) − GlcSO_3_Na (C_6_H_10_O_8_SNa) + H]^+^, 609.1 [M_Na_ + Na − MeGlcSO_3_Na (C_7_H_12_O_8_SNa) − Glc (C_6_H_10_O_5_) − Qui (C_6_H_10_O_4_) − GlcSO_3_Na (C_6_H_10_O_8_SNa) + H]^+^, 481.1 [M_Na_ + Na − Agl (C_30_H_45_O_2_) − MeGlcSO_3_Na (C_7_H_12_O_8_SNa) − GlcSO_3_Na (C_6_H_10_O_8_SNa) + H]^+^ (Figure S16).

### 3.4. Solvolytic Desulfation of Glacialisoside B (***2***) and Isolation of Derivatives

Glacialisoside B (**2**) (5 mg) was dissolved in 1 mL of a mixture of absolute pyridine and dioxane (1:1) and exposed at room temperature for 2 h. The reaction mixture was concentrated in vacuo and then separated in a silica gel mini-column, using a stepped gradient of solvent systems CHCl_3_/EtOH/H_2_O in ratios: 10:7.5:1 → 10:7.5:1/10:10:1.7 (1/1 *v*/*v*) → 10:10:1.7. As a result, three desulfated derivatives were isolated, namely, 1.1 mg of glacialisoside A (**1**), 0.8 mg of DS-glacialisoside B (**3**) and 0.9 mg of di-DS-glacialisoside B (**4**), as well as glacialisoside B (**2**) (0.3 mg).

#### 3.4.1. Glacialisoside A (**1**) Obtained as a Result of Solvolytic Desulfation

(−)HR-ESI-MS *m*/*z*: 1311.5697 (calc. 1311.5685) [M_Na_ − Na]^−^; (+)HR-ESI-MS *m*/*z*: 1357.5460 (calc. 1357.5470) [M_Na_ + Na]^+^; (*+*)HR-ESI-MS/MS *m*/*z*: 1237.6 [M_Na_ + Na − NaHSO_4_]^+^, 1079.5 [M_Na_ + Na − MeGlcSO_3_Na (C_7_H_12_O_8_SNa) + H]^+^, 921.2 [M_Na_ + Na − Agl (C_30_H_45_O_2_) + H]^+^, 759.2 [M_Na_ + Na − Agl (C_30_H_45_O_2_) − Glc (C_6_H_10_O_5_) + H]^+^, 609.1 [M_Na_ + Na − MeGlcSO_3_Na (C_7_H_12_O_8_SNa) − Glc (C_6_H_10_O_5_) − Qui (C_6_H_10_O_4_) − Glc (C_6_H_10_O_5_) + H]^+^, 481.1 [M_Na_ + Na − Agl (C_30_H_45_O_2_) − MeGlcSO_3_Na (C_7_H_12_O_8_SNa) − Glc (C_6_H_10_O_5_) + H]^+^ (Figure S17).

#### 3.4.2. DS-Glacialisoside B (**3**)

(−)HR-ESI-MS *m*/*z*: 1311.5678 (calc. 1311.5685) [M_Na_ − Na]^−^; (+)HR-ESI-MS *m*/*z*: 1357.5458 (calc. 1357.5470) [M_Na_ + Na]^+^; (*+*)HR-ESI-MS/MS *m*/*z*: 1237.6 [M_Na_ + Na − NaHSO_4_]^+^, 1093.5 [M_Na_ + Na − GlcSO_3_Na (C_6_H_10_O_8_SNa) + H]^+^, 921.2 [M_Na_ + Na − Agl (C_30_H_45_O_2_) + H]^+^, 801.3 [M_Na_ + Na − Agl (C_30_H_45_O_2_) − NaSO_4_]^+^, 657.2 [M_Na_ + Na − Agl (C_30_H_45_O_2_) − GlcSO_3_Na (C_6_H_10_O_8_SNa) + H]^+^, 507.2 [M_Na_ + Na − MeGlc (C_7_H_13_O_6_) − Glc (C_6_H_10_O_5_) − Qui (C_6_H_10_O_4_) − GlcSO_3_Na (C_6_H_10_O_8_SNa) + H]^+^; (−)HR-ESI-MS/MS *m*/*z*: 1135.5 [M_Na_ − Na − MeGlc (C_7_H_13_O_6_) − H]^−^, 973.4 [M_Na_ − Na − MeGlc (C_7_H_13_O_6_) − Glc (C_6_H_10_O_5_) − H]^−^, 827.3 [M_Na_ − Na − MeGlc (C_7_H_13_O_6_) − Glc (C_6_H_10_O_5_) − Qui (C_6_H_10_O_4_) − H]^−^ (Figure S18).

#### 3.4.3. di-DS-Glacialisoside B (**4**)

(−)HR-ESI-MS *m*/*z*: 1231.6117 (calc. 1231.6117) [M – H]^−^; (+)HR-ESI-MS *m*/*z*: 1255.6076 (calc. 1255.6080) [M + Na]^+^; (*+*)HR-ESI-MS/MS *m*/*z*: 1079.5 [M + Na − MeGlc (C_7_H_13_O_6_) + H]^+^, 917.5 [M + Na − MeGlc (C_7_H_13_O_6_) − Glc (C_6_H_10_O_5_) + H]^+^, 819.3 [M + Na − Agl (C_30_H_45_O_2_) + H]^+^, 771.4 [M + Na − MeGlc (C_7_H_13_O_6_) − Glc (C_6_H_10_O_5_) − Qui (C_6_H_10_O_4_) + H]^+^, 657.2 [M + Na − Agl (C_30_H_45_O_2_) − Glc (C_6_H_10_O_5_) + H]^+^, 481.1 [M + Na − Agl (C_30_H_45_O_2_) − MeGlc (C_7_H_13_O_6_) − Glc (C_6_H_10_O_5_)+ H]^+^; (−)HR-ESI-MS/MS *m*/*z*: 1055.5 [M − H − MeGlc (C_7_H_13_O_6_)+ H]^−^, 893.5 [M − H − MeGlc (C_7_H_13_O_6_) − Glc (C_6_H_10_O_5_)+ H]^−^, 731.4 [M − H − MeGlc (C_7_H_13_O_6_) − Glc (C_6_H_10_O_6_) − Qui (C_6_H_10_O_4_)+ H]^−^ (Figure S19).

### 3.5. Hemolytic Activity

Erythrocytes were isolated from a human blood (B(III) Rh+) by centrifuging three times for 5 min (450 g) with phosphate-buffered saline (PBS, pH 7.4) at 4 °C using a LABOFUGE 400R centrifuge (Heraeus, Hanau, Germany). Ice-cold PBS was used to resuspend the erythrocyte residue to a final optical density of 1.5 at 700 nm. Subsequently, 10 µL of the tested compound solutions or control (cucumarioside A_0_-1) were added to 90 µL of the erythrocyte suspension in V-bottom 96-well plates and incubated for 1 h at 37 °C. Following centrifugation at 900× *g* for 10 min on an LMC-3000 laboratory centrifuge (Biosan, Riga, Latvia), 70 µL of the supernatant was transferred into new flat-bottom plates. Erythrocyte lysis was quantified by measuring the hemoglobin concentration in the supernatant, using a Synergy H1 microplate reader (BioTek Instruments Inc., Winooski, VT, USA) at λ = 570 nm. The effective dose causing 50% erythrocyte lysis (ED_50_) was calculated using SigmaPlot software (version 14, Grafiti LLC, Palo Alto, CA, USA). All experiments were performed twice in triplicate (*p* < 0.05).

### 3.6. Cytotoxic Activity (MTT Assay)

To evaluate cytotoxicity, five cancer cell lines (MCF-7, T-47D, MDA-MB-231, MDA-MB-468, and PANC-1) and a non-malignant human breast epithelial cell line (MCF-10A) were cultured in 96-well plates (6 × 10^3^ cells/well) and allowed to adhere for 24 h. Cells between passages 3 and 8 were utilized for the MTT assay. The cells were then treated with compounds **1**–**4** at concentrations ranging from 0.07 to 20 μM. Cucumarioside A_0_-1 (Cuc A_0_-1) was used at the same concentrations as a positive control. After 24 h of incubation, the medium was replaced with 100 μL of fresh medium and 10 μL (5 mg/mL) of MTT (3-(4,5-dimethylthiazol-2-yl)-2,5-diphenyltetrazolium bromide) solution (neoFroxx, D-Einhausen, Germany) was added. After 4 h of incubation, 100 μL of SDS-HCl solution (1 g SDS/10 mL d-H_2_O/17 μL 6 N HCl) was added, and the plates were kept in a CO_2_ incubator overnight. Absorbance was recorded at 570 nm using a Synergy H1 microplate reader (BioTek Instruments Inc., Winooski, VT, USA). The cytotoxic activity of the studied glycosides was assessed by the IC_50_ values, defined as the concentration inhibiting 50% of cellular metabolic activity. The experiments were conducted in triplicate, *p* < 0.05.

### 3.7. Colony Formation Assay

The clonogenic assay was performed using a slightly modified version of a previously described method [42]. MDA-MB-231, MDA-MB-468, MCF-7, and PANC-1 cells were seeded into 6-well plates (0.3 × 10^2^ cells/mL) for 24 h to allow adhesion and then treated with the investigated compounds (0.2, 0.5, 1, and 2 µM). Cells between passages 3 and 8 were utilized for the clonogenic assay. After a 10–14-day incubation period in a CO_2_ incubator, the resulting colonies (≥50 cells) were fixed with methanol and stained with 0.5% crystal violet for 25 min each. After washing and air-drying, colonies were quantified using the BIO-PRINT-Cx4 system with Bio-Vision software (v. 18.01; Vilber, Paris, France) according to the manufacturer’s instructions. Data are presented as the percentage of colony growth inhibition compared to the control. The experiments were conducted in triplicate, *p* < 0.05.

## Figures and Tables

**Table 1:** One- and two-dimensional NMR data of the aglycone moieties of glacialisoside A (**1**).

Position	*δ*_C,_ Mult. ^a^	*δ*_H,_ Mult. (*J* in Hz) ^b^	HMBC	ROESY
1	36.0 CH_2_	1.35 m		H-3, H-5, H-11, H-19
2	26.9 CH_2_	2.00 m		
		1.83 m		H-19, H-30
3	89.2 CH	3.19 dd (11.8; 3.5)		H-1, H-5, H-31, H-1 Xyl1
4	39.3 C			
5	48.0 CH	0.94 dd (11.1; 4.27)	C: 1, 4, 10	H-1, H-3, H-19, H-31
6	23.2 CH_2_	1.95 m		
7	119.8 CH	5.68 m		H-15, H-32
8	146.6 C			
9	47.3 CH	3.36 brd (14.6)		H-19
10	35.4 C			
11	22.8 CH_2_	1.72 m		H-1
		1.48 m		
12	30.2 CH_2_	2.00 m		H-17, H-21
13	58.7 C			
14	51.2 C			
15	34.1 CH_2_	1.78 m		H-7, H-32
		1.52 m		
16	24.3 CH_2_	1.93 m		H-32
		1.75 m		
17	53.0 CH	2.20 dd (9.7; 4.2)	C: 13, 18, 21	H-12, H-21, H-23, H-32
18	180.8 C			
19	23.9 CH_3_	1.12 s	C: 1, 9, 10	H-1, H-2, H-6, H-9, H-30
20	84.7 C			
21	26.0 CH_3_	1.40 s	C: 17, 20, 22	H-12, H-17
22	38.5 CH_2_	1.61 m		H-24
		1.55 m		H-24
23	21.8 CH_2_	1.37 m		
		1.30 m		H-17
24	37.7 CH_2_	1.92 m	C: 25	H-22, H-26
25	145.3 C			
26	110.8 CH_2_	4.75 brs	C: 24, 27	
		4.71 brs	C: 24, 27	
27	22.0 CH_3_	1.66 s	C: 24, 25, 26	
30	17.2 CH_3_	1.01 s	C: 3, 4, 5, 31	H-2, H-6, H-19, H-31
31	28.6 CH_3_	1.18 s	C: 3, 4, 5, 30	H-3, H-5, H-6, H-30, H-1 Xyl1
32	30.7 CH_3_	1.13 s	C: 8, 13, 14, 15	H-7, H-15, H-16, H-17

^a^ Recorded at 176.04 MHz in C_5_D_5_N/D_2_O (4/1). ^b^ Recorded at 700.13 MHz in C_5_D_5_N/D_2_O (4/1). The original spectra of **1** and **2** are provided in Figures S1–S6.

**Table 2:** One- and two-dimensional NMR data of carbohydrate moiety of glacialisoside A (**1**).

Atom	*δ*_C_ Mult. ^a,b,c^	*δ*_H_ Mult. (*J* in Hz) ^d^	HMBC	ROESY
Xyl1 (1→C-3)				
1	104.8 CH	4.67 d (7.3)	C: 3; C: 1 Xyl1	H-3; H-3, 5 Xyl1
2	**82.7** CH	3.93 t (8.4)	C: 1, 3 Xyl1	H-1 Qui2; H-4 Xyl1
3	75.2 CH	4.16 t (8.4)	C: 2, 4 Xyl1	H-1, 5 Xyl1
4	**77.4** CH	4.21 m	C: 3 Xyl1; C: 1 Glc5	H-2 Xyl1; H-1 Glc5
5	63.5CH_2_	4.38 dd (11.5; 5.3)	C: 1, 3, 4 Xyl1	
		3.64 brt (10.5)	C: 1 Xyl1	H-1, 3 Xyl1
Qui2 (1→2Xyl1)				
1	104.8 CH	4.97 d (7.7)	C: 2 Xyl1	H-2 Xyl1; H-5 Qui2
2	75.5 CH	3.87 t (8.5)	C: 1, 3 Qui2	H-4 Qui2
3	75.2 CH	3.95 t (8.5)	C: 2, 4 Qui2	H-1, 5 Qui2
4	**86.4** CH	3.55 t (8.5)	C: 3, 5, 6 Qui2; C: 1 Glc3	H-1 Glc3; H-2 Qui2
5	71.5 CH	3.64 m	C: 4 Qui2	H-1, 3 Qui2
6	17.9 CH_3_	1.61 d (6.0)	C: 4, 5 Qui2	H-4, 5 Qui2
Glc3 (1→4Qui2)				
1	104.0 CH	4.86 d (7.5)	C: 4 Qui2	H-4 Qui2; H-3, 5 Glc3
2	73.5 CH	3.91 t (8.9)	C: 1, 3 Glc3	
3	**87.5** CH	4.12 t (8.9)	C: 4 Glc3, C: 1 MeGlc4	H-1 MeGlc4; H-1 Glc3
4	69.4 CH	3.84 t (8.9)	C: 3, 5, 6 Glc3	
5	77.1 CH	3.91 t (8.9)	C: 4 Glc3	H-1 Glc3
6	61.6 CH_2_	4.33 brd (11.7)		
		4.02 dd (11.7; 5.7)	C: 5 Glc3	
MeGlc4 (1→3Glc3)				
1	104.8 CH	5.12 d (8.1)	C: 3 Glc3	H-3 Glc3; H-3, 5 MeGlc4
2	74.3 CH	3.79 t (8.9)	C: 1, 3 MeGlc4	H-4 MeGlc4
3	86.4 CH	3.64 t (8.9)	C: 2, 4 MeGlc4; OMe	H-1, 5 MeGlc4
4	69.9 CH	3.96 t (8.9)	C: 5, 6 MeGlc4	H-2 MeGlc4
5	75.6 CH	4.04 t (8.9)	C: 4 MeGlc4	H-1, 3 MeGlc4
6	*67.1* CH_2_	4.96 brdd (11.3; 9.7)	C: 4 MeGlc4	
		4.72 dd (11.3; 5.7)	C: 5 MeGlc4	
OMe	60.5 CH_3_	3.76 s	C: 3 MeGlc4	
Glc5 (1→4Xyl1)				
1	102.7 CH	4.88 d (7.7)	C: 4 Xyl1	H-4 Xyl1; H-3, 5 Glc5
2	74.0 CH	3.88 t (8.8)	C: 1, 3 Glc5	
3	77.3 CH	4.13 t (8.8)	C: 2, 4 Glc5	H-5 Glc5
4	71.0 CH	3.93 t (8.8)	C: 5, 6 Glc5	
5	77.7 CH	3.88 t (8.8)	C: 4 Glc5	H-1, 3 Glc5
6	61.9 CH_2_	4.36 brd (12.1)	C: 4 Glc5	
		4.07 dd (12.1; 6.6)	C: 5 Glc5	

^a^ Recorded at 176.04 MHz in C_5_D_5_N/D_2_O (4/1). ^b^ Bold = interglycosidic positions. ^c^ Italic = sulfate positions. ^d^ Recorded at 700.13 MHz in C_5_D_5_N/D_2_O (4/1). Multiplicity by 1D TOCSY. The original spectra of **1** are provided in Figures S1–S7.

**Table 3:** One- and two-dimensional NMR data of carbohydrate moiety of glacialisoside B (**2**).

Atom	*δ*_C_ Mult. ^a,b,c^	*δ*_H_ Mult. (*J* in Hz) ^d^	HMBC	ROESY
Xyl1 (1→C-3)				
1	104.8 CH	4.68 d (7.5)	C: 3; C: 1 Xyl1	H-3; H-3, 5 Xyl1
2	**82.7** CH	3.94 t (8.7)	C: 1 Xyl1; C: 1 Qui2	H-1 Qui2
3	75.2 CH	4.16 t (8.7)	C: 2, 4 Xyl1	H-1, 5 Xyl1
4	**77.7** CH	4.21 m	C: 3 Xyl1; C: 1 Glc5	H-2 Xyl1; H-1 Glc5
5	63.5CH_2_	4.39 dd (12.4; 5.0)	C: 1, 3, 4 Xyl1	
		3.65 brdd (12.4; 8.7)		H-1, 3 Xyl1
Qui2 (1→2Xyl1)				
1	104.8 CH	4.99 d (8.4)	C: 2 Xyl1	H-2 Xyl1; H-3,5 Qui2
2	75.6 CH	3.87 t (9.2)	C: 1, 3 Qui2	H-4 Qui2
3	75.2 CH	3.95 t (9.2)	C: 2, 4 Qui2	H-1 Qui2
4	**86.4** CH	3.55 t (9.2)	C: 3, 5, 6 Qui2; C: 1 Glc3	H-1 Glc3; H-2 Qui2
5	71.5 CH	3.64 dd (9.2; 6.1)		H-1 Qui2
6	17.9 CH_3_	1.62 d (6.1)	C: 4, 5 Qui2	H-4, 5 Qui2
Glc3 (1→4Qui2)				
1	104.0 CH	4.86 d (8.0)	C: 4 Qui2	H-4 Qui2; H-3, 5 Glc3
2	73.5 CH	3.91 t (8.5)	C: 1, 3 Glc3	
3	**87.6** CH	4.12 t (8.5)	C: 2, 4 Glc3, C: 1 MeGlc4	H-1 MeGlc4; H-1 Glc3
4	69.3 CH	3.84 t (8.5)	C: 3, 5, 6 Glc3	
5	77.1 CH	3.91 t (8.5)	C: 6 Glc3	H-1, 3 Glc3
6	61.7 CH_2_	4.34 brd (11.1)		
		4.02 dd (11.1; 5.3)	C: 5 Glc3	
MeGlc4 (1→3Glc3)				
1	104.8 CH	5.12 d (7.9)	C: 3 Glc3	H-3 Glc3; H-3, 5 MeGlc4
2	74.3 CH	3.79 t (8.6)	C: 1, 3 MeGlc4	
3	86.4 CH	3.65 t (8.6)	C: 2, 4 MeGlc4; OMe	H-1, 5 MeGlc4
4	69.9 CH	3.98 t (8.6)	C: 3, 5, 6 MeGlc4	
5	75.5 CH	4.04 m		H-1, 3 MeGlc4
6	*67.0* CH_2_	4.96 d (11.5)		
		4.73 brdd (11.5; 4.3)	C: 5 MeGlc4	
OMe	60.5 CH_3_	3.76 s	C: 3 MeGlc4	
Glc5 (1→4Xyl1)				
1	102.4 CH	4.91 d (7.7)	C: 4 Xyl1	H-4 Xyl1; H-3, 5 Glc5
2	72.9 CH	3.92 t (8.4)	C: 1, 3 Glc5	
3	*84.4* CH	5.03 t (8.4)	C: 2, 4 Glc5	H-1 5 Glc5
4	69.9 CH	3.98 t (8.4)	C: 3, 5, 6 Glc5	
5	77.7 CH	3.86 t (8.4)		H-1, 3 Glc5
6	61.6 CH_2_	4.31 brd (12.6)		
		4.02 dd (12.6; 6.3)		

^a^ Recorded at 176.04 MHz in C_5_D_5_N/D_2_O (4/1). ^b^ Bold = interglycosidic positions. ^c^ Italic = sulfate positions. ^d^ Recorded at 700.13 MHz in C_5_D_5_N/D_2_O (4/1). Multiplicity by 1D TOCSY. The original spectra of **2** are provided in Figures S9–S15.

**Table 4:** The cytotoxic activities of compounds **1**–**4** and cucumarioside A_0_-1 (positive control) against human erythrocytes and MCF-10A, MCF-7, T-47D, MDA-MB-231, MDA-MB-468, and PANC-1 human cell lines.

Glycosides	ED_50_, µM, Erythrocytes	Cytotoxicity, IC_50_ µM					
		MCF-10A	MCF-7	T-47D	MDA-MB-231	MDA-MB-468	PANC-1
glacialisoside A (**1**)	0.25 ± 0.03	18.86 ± 1.37	>20.0	16.33 ± 0.47	8.20 ± 0.21	>20.0	17.69 ± 0.65
glacialisoside B (**2**)	0.50 ± 0.01	>20.0	14.80 ± 0.64	16.21 ± 0.50	8.09 ± 0.16	15.16 ± 0.17	14.74 ± 0.36
DS-glacialisoside B-2 (**3**)	1.81 ± 0.06	>20.0	>20.0	>20.0	>20.0	>20.0	18.58 ± 1.12
di-DS-glacialisoside B (**4**)	5.54 ± 0.24	>20.0	>20.0	>20.0	>20.0	>20.0	>20.0
cucumarioside A_0_-1	1.05 ± 0.15	12.48 ± 0.87	13.30 ± 1.26	14.16 ± 1.53	5.58 ± 0.78	5.02 ± 0.54	5.23 ± 0.48

**Table 5:** Cancer cell selectivity index (SI; a ratio of IC_50_ calculated for erythrocytes and cancer cells) of compounds **1**–**4** and cucumarioside A_0_-1 (positive control), relative to hemolytic activity.

Glycosides	Selectivity Index (SI) Relative to Hemolytic Activity					
	MCF-10A	MCF-7	T-47D	MDA-MB-231	MDA-MB-468	PANC-1
glacialisoside A (**1**)	0.013	>0.013	0.015	0.03	>0.013	0.014
glacialisoside B (**2**)	>0.025	0.034	0.031	0.062	0.033	0.034
DS-glacialisoside B-2 (**3**)	>0.09	>0.09	>0.09	>0.09	>0.09	0.097
di-DS-glacialisoside B (**4**)	>0.227	>0.227	>0.227	>0.227	>0.227	>0.227
cucumarioside A_0_-1	0.084	0.079	0.074	0.19	0.21	0.20

**Table 6:** The inhibition of colony formation in MDA-MB-231, MDA-MB-468, MCF-7, and PANC-1 human cell lines by glacialisoside A (**1**) and cucumarioside A_0_-1 (positive control).

Glycosides	Colony Formation, IC_50_, µM			
	MDA-MB-231	MDA-MB-468	MCF-7	PANC-1
glacialisoside A (**1**)	0.47	<0.20	0.92	1.28
cucumarioside A_0_-1	0.29	0.32	0.60	0.40

**Table 7:** Colony formation selectivity index (SI) against cancer cell lines relative to hemolytic activity by glacialisoside A (**1**) and cucumarioside A_0_-1 (positive control).

Glycosides	Selectivity Index (SI) Relative to Hemolytic Activity			
	MDA-MB-231	MDA-MB-468	MCF-7	PANC-1
glacialisoside A (**1**)	0.53	>1.25	0.27	0.20
cucumarioside A_0_-1	3.62	3.28	1.75	2.63

## Data Availability

The original contributions presented in this study are included in the article. Further inquiries can be directed to the corresponding author.

## Supplementary Materials

The following supporting information can be downloaded at https://www.mdpi.com/article/10.3390/md24080269/s1.
