# Supplementary material for: Triterpene Glycosides from the Sea Cucumber Ocnus glacialis Display Cytotoxic and Colony-Inhibiting Activity Against Cancer Cells
Source: Mar Drugs. 2026 Aug 3;24(8):269. doi: 10.3390/md24080269 (PMC13514455; doi:10.3390/md24080269)
Supplement: Supplementary file 1 [file marinedrugs-24-00269-s001.zip › marinedrugs-4423619-supplementary.pdf]

Supplementary data content page

Title: Triterpene Glycosides from the Sea Cucumber *Ocnus glacialis* Display Cytotoxic and Colony-Inhibiting Activity Against Cancer Cells

Authors: Alexandra S. Silchenko [1,*], Ekaterina A. Chingizova [1], Ekaterina S. Menchinskaya [1], Kseniya M. Tabakmakher [1], Anatoly I. Kalinovsky [1], Sergey A. Avilov [1], Roman S. Popov [1], Pavel S. Dmitrenok [1] and Vladimir I. Kalinin [1]

Address: [1]*G.B. Elyakov Pacific Institute of Bioorganic Chemistry, Far Eastern Branch of Russian Academy of Sciences, Pr. 100-let Vladivostoka 159, 690022 Vladivostok, Russia*

Correspondence: silchenko_als@piboc.dvo.ru; Tel.: +7-423-231-1168

Content:

Figure S1. The ^{13}C NMR (176.04 MHz) spectrum of glacialisoside A (**1**) in C_5D_5N/D_2O (4/1)

Figure S2. The ^{1}H NMR (700.13 MHz) spectrum of glacialisoside A (**1**) in C_5D_5N/D_2O (4/1)

Figure S3. The COSY (700.13 MHz) spectrum of glacialisoside A (**1**) in C_5D_5N/D_2O (4/1)

Figure S4. The HSQC (700.13 MHz) spectrum of glacialisoside A (**1**) in C_5D_5N/D_2O (4/1)

Figure S5. The ROESY (700.13 MHz) spectrum of glacialisoside A (**1**) in C_5D_5N/D_2O (4/1)

Figure S6. The HMBC (700.13 MHz) spectrum of glacialisoside A (**1**) in C_5D_5N/D_2O (4/1)

Figure S7. 1 D TOCSY (700.13 MHz) spectra of Xyl1, Qui2, Glc3, MeGlc4, Glc5 of glacialisoside A (**1**) in C_5D_5N/D_2O (4/1)

Figure S8. HR-ESI-MS and ESI-MS/MS spectra of glacialisoside A (**1**)

Figure S9. The ^{13}C NMR (176.04 MHz) spectrum of glacialisoside B (**2**) in C_5D_5N/D_2O (4/1)

Figure S10. The ^{1}H NMR (700.13 MHz) spectrum of glacialisoside B (**2**) in C_5D_5N/D_2O (4/1)

Figure S11. The COSY (700.13 MHz) spectrum of glacialisoside B (**2**) in C_5D_5N/D_2O (4/1)

Figure S12. The HSQC (700.13 MHz) spectrum of glacialisoside B (**2**) in C_5D_5N/D_2O (4/1)

Figure S13. The ROESY (700.13 MHz) spectrum of glacialisoside B (**2**) in C_5D_5N/D_2O (4/1)

Figure S14. The HMBC (700.13 MHz) spectrum of glacialisoside B (**2**) in C_5D_5N/D_2O (4/1)

Figure S15. 1 D TOCSY (700.13 MHz) spectra of Xyl1, Qui2, Glc3, MeGlc4, Glc5 of glacialisoside B (**2**) in C_5D_5N/D_2O (4/1)

Figure S16. HR-ESI-MS and ESI-MS/MS spectra of glacialisoside B (**2**)

Figure S17. HR-ESI-MS and ESI-MS/MS spectra of **1** obtained as a result of solvolytic desulfation

Figure S18. HR-ESI-MS and ESI-MS/MS spectra of DS-glacialisoside B (**3**)

Figure S19. HR-ESI-MS and ESI-MS/MS spectra of di-DS-glacialisoside B (**4**)

Table S1. One- and two-dimensional NMR data of aglycone moiety of glacialisoside B (**2**)

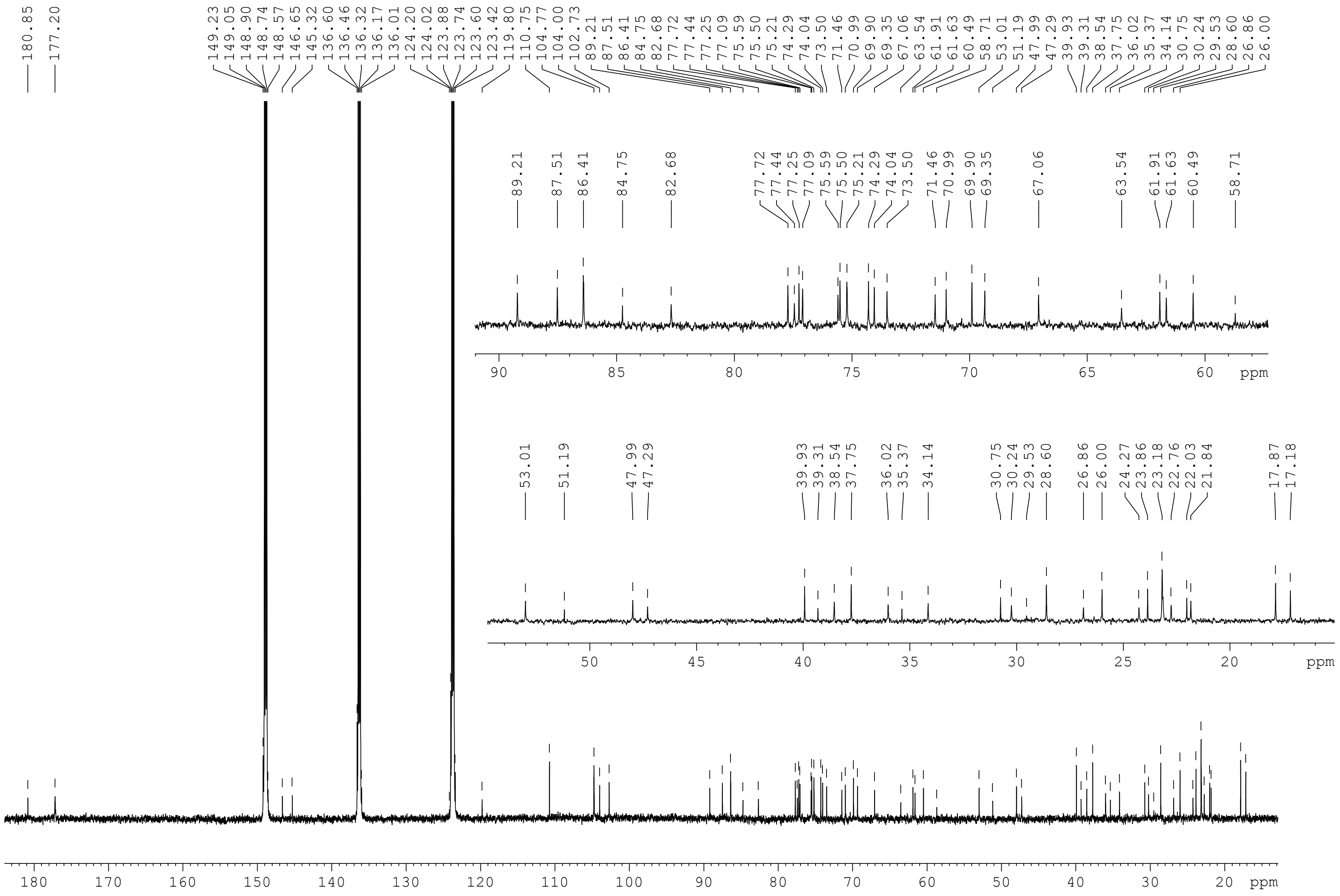

Figure S1. The ^{13}C NMR (176.04 MHz) spectrum of glacialisoside A (**1**) in C_5D_5N/D_2O (4/1)

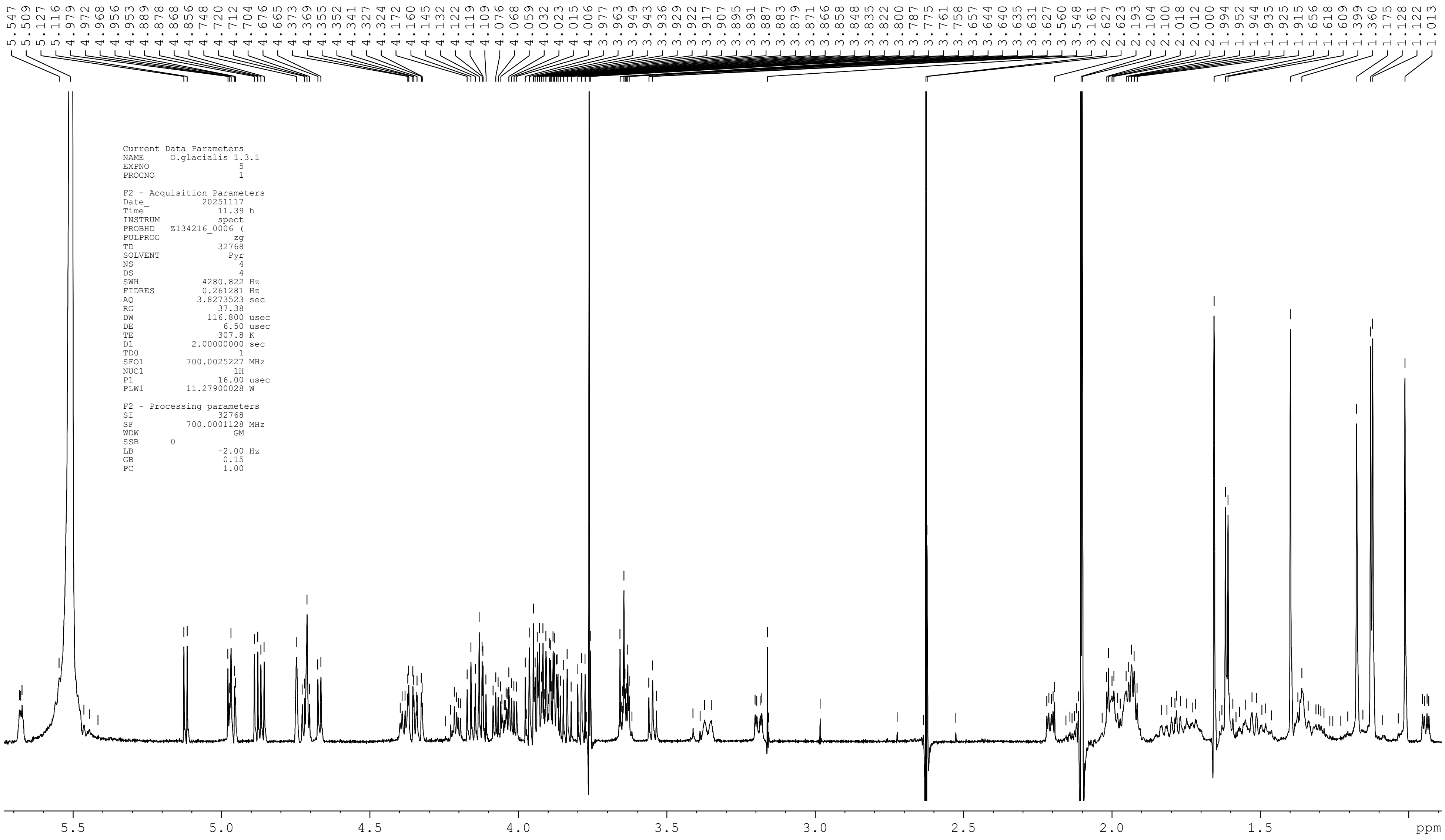

Figure S2. The ^{1}H NMR (700.13 MHz) spectrum of glacialisoside A (**1**) in C_5D_5N/D_2O (4/1)

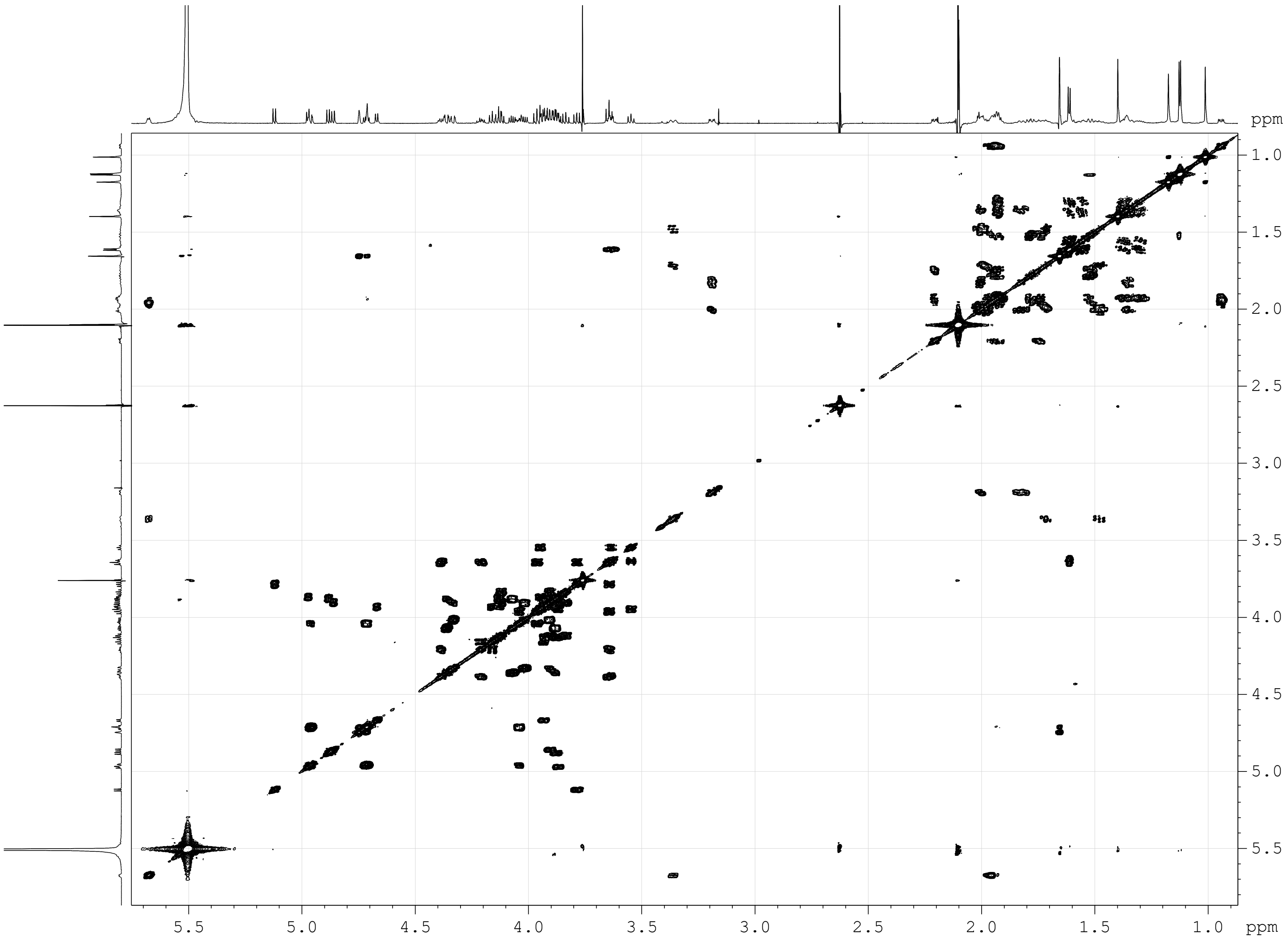

Figure S3. The COSY (700.13 MHz) spectrum of glacialisoside A (**1**) in C_5D_5N/D_2O (4/1)

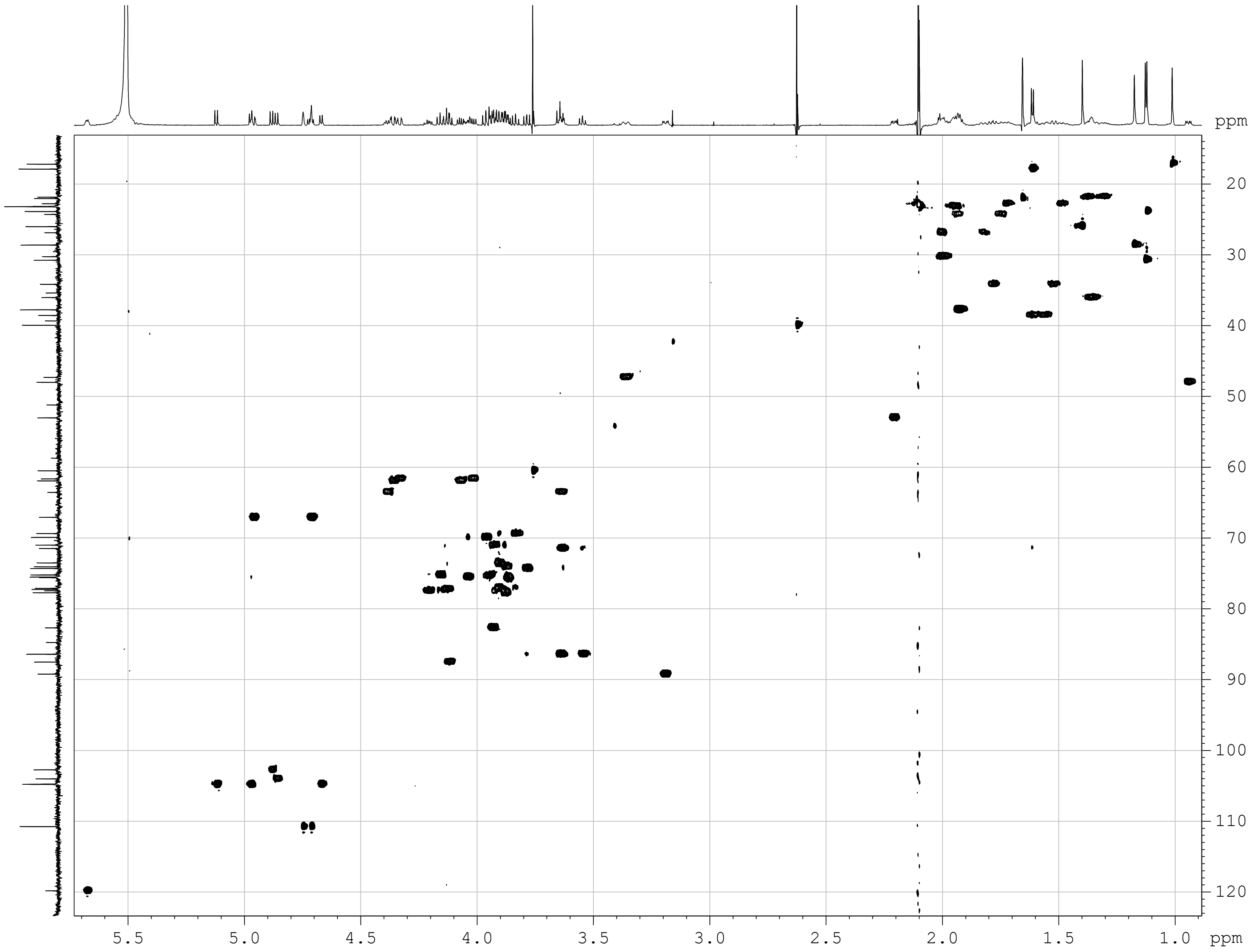

Figure S4. The HSQC (700.13 MHz) spectrum of glacialisoside A (**1**) in C_5D_5N/D_2O (4/1)

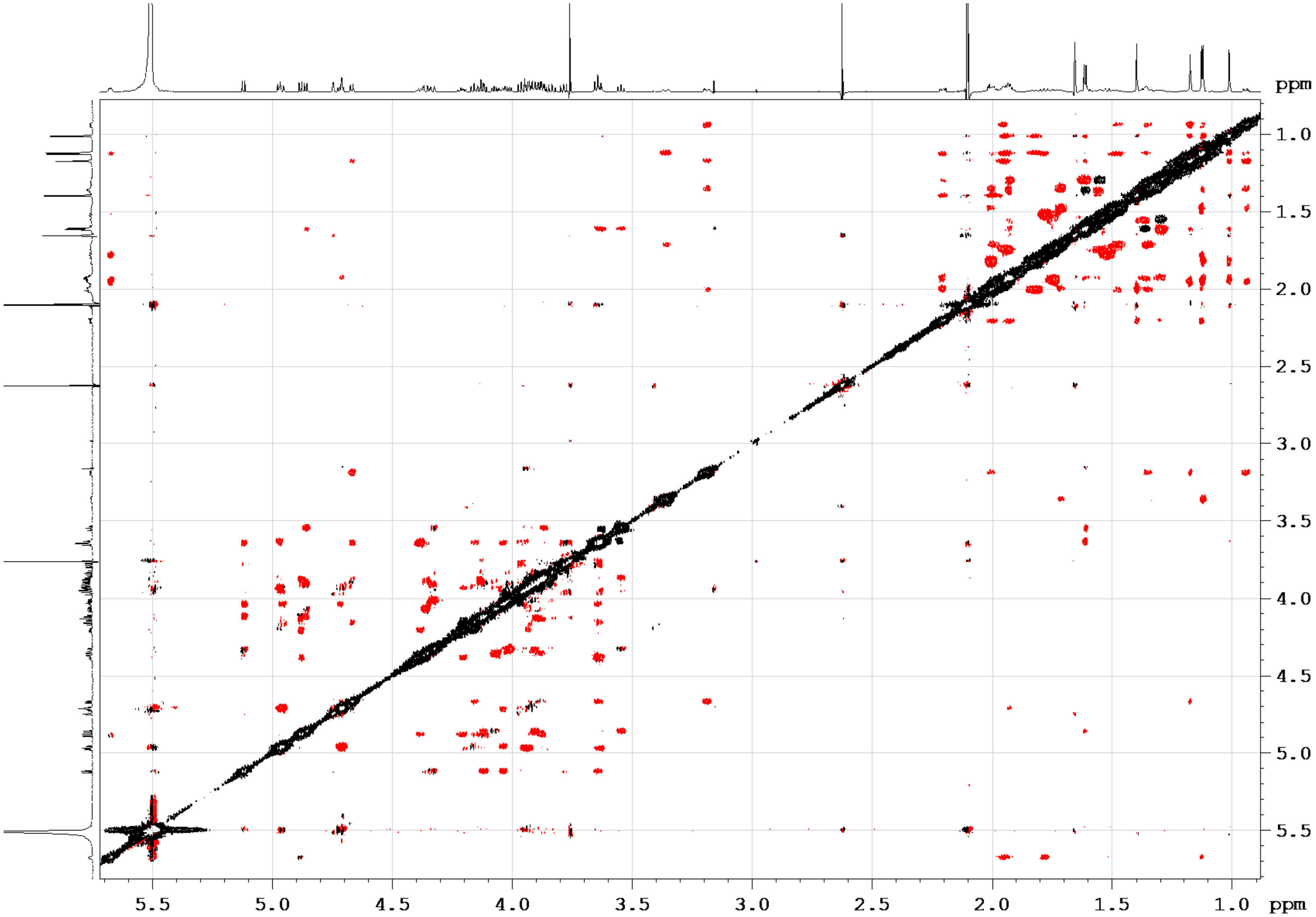

Figure S5. The ROESY (700.13 MHz) spectrum of glacialisoside A (**1**) in C_5D_5N/D_2O (4/1)

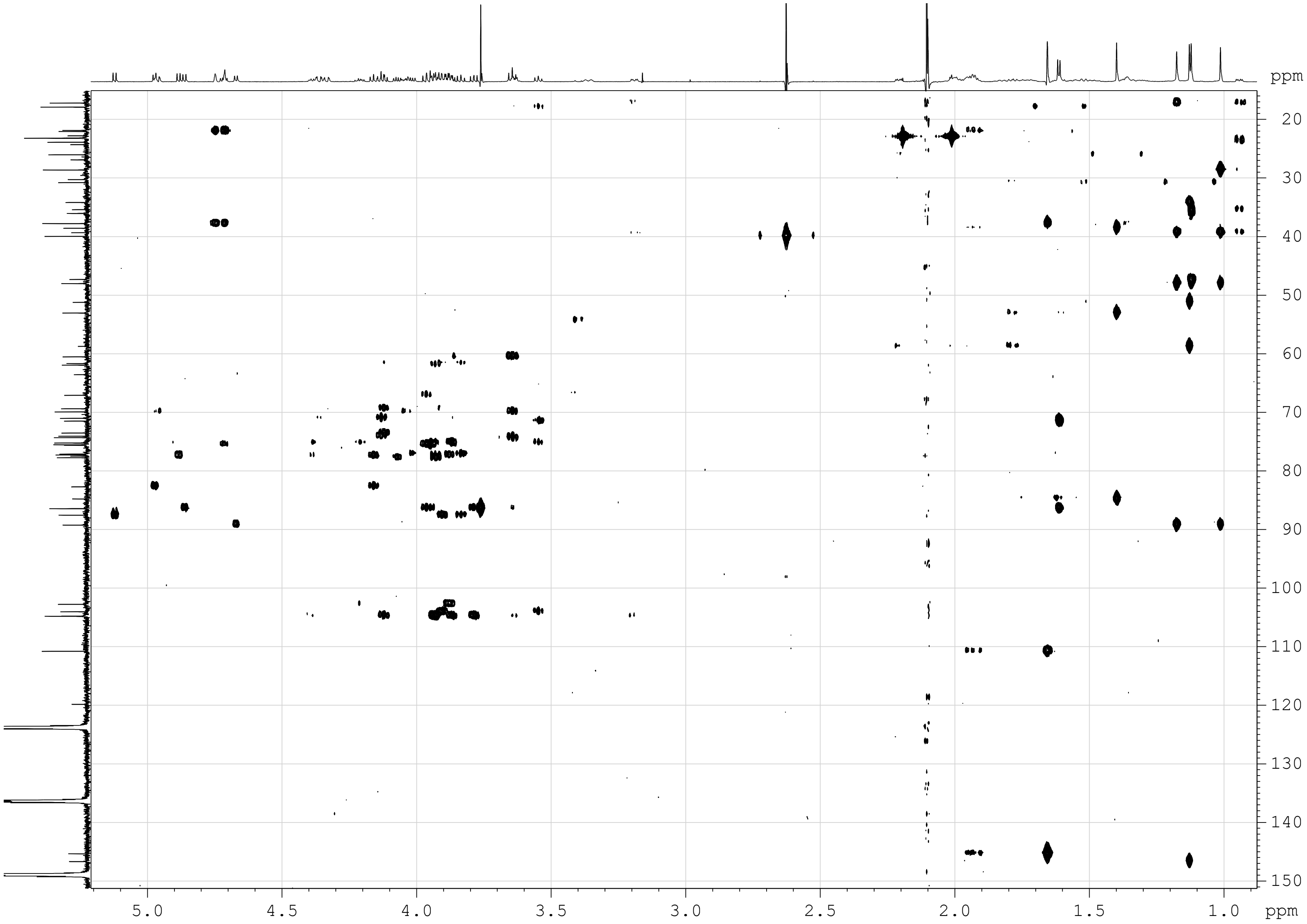

Figure S6. The HMBC (700.13 MHz) spectrum of glacialisoside A (**1**) in C_5D_5N/D_2O (4/1)

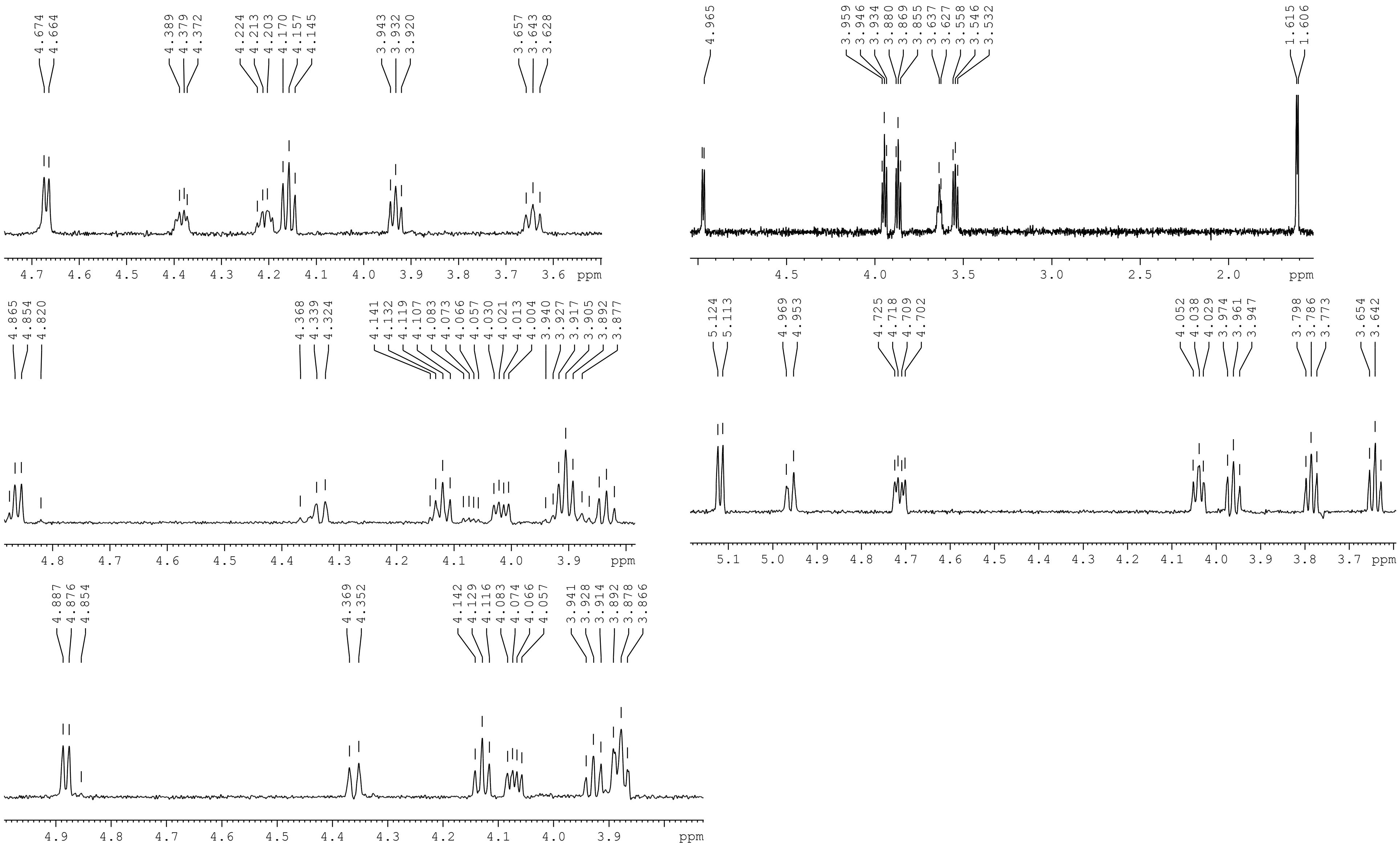

Figure S7. 1 D TOCSY (700.13 MHz) spectra of Xyl1, Qui2, Glc3, MeGlc4, Glc5 of glacialisoside A (**1**) in C_5D_5N/D_2O (4/1)

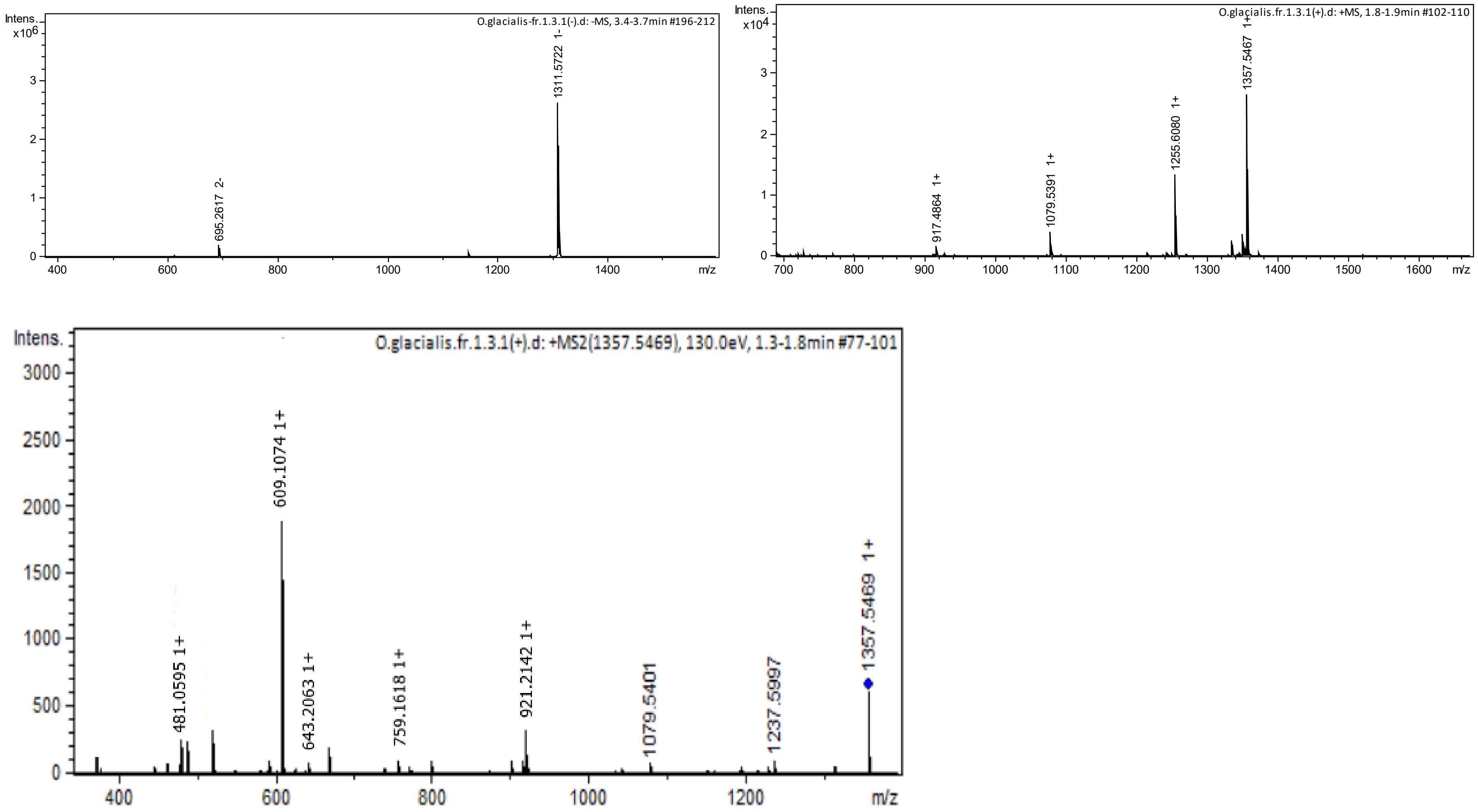

Figure S8. HR-ESI-MS and ESI-MS/MS spectra of glacialisoside A (**1**)

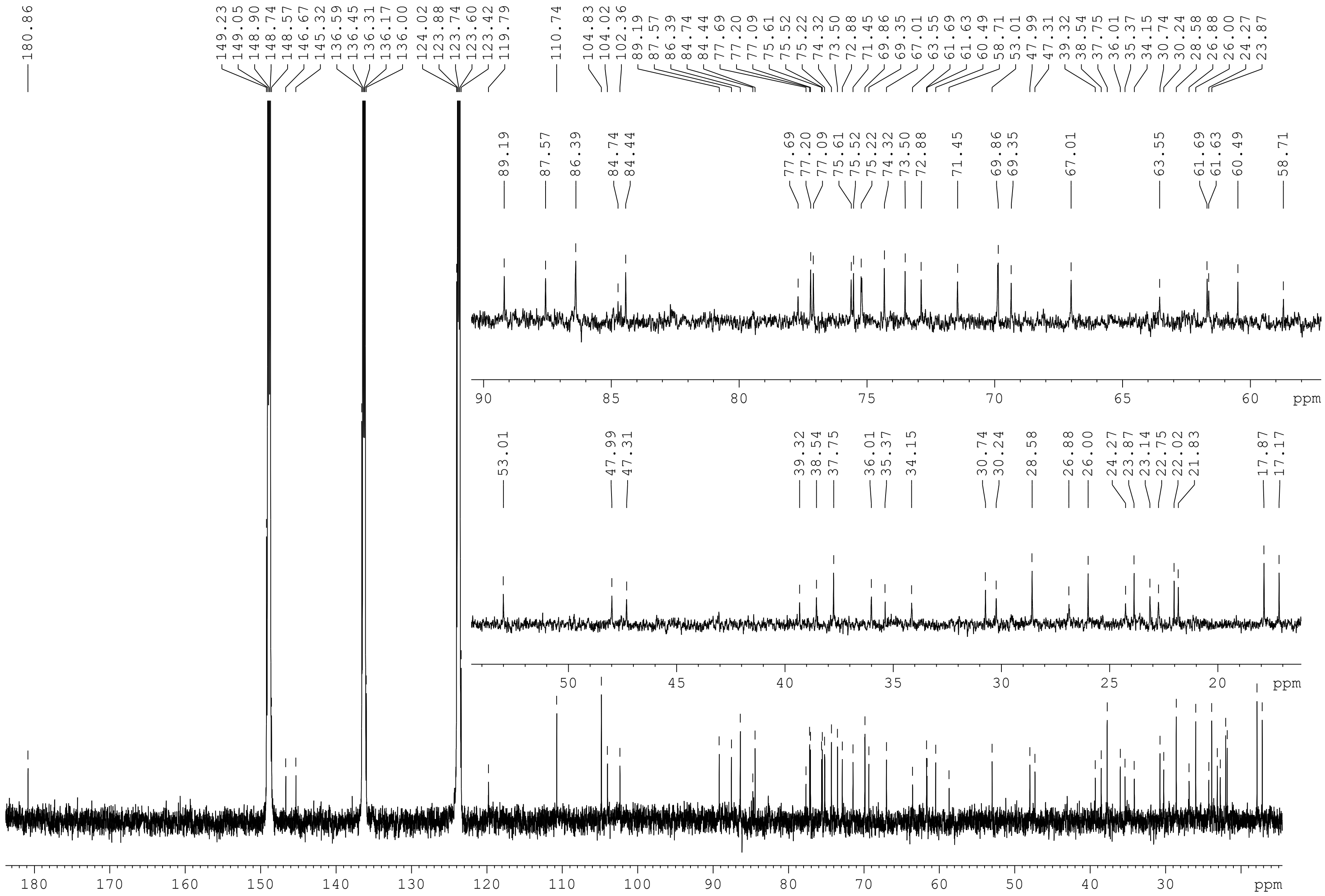

Figure S9. The ^{13}C NMR (176.04 MHz) spectrum of glacialisoside B (**2**) in C_5D_5N/D_2O (4/1)

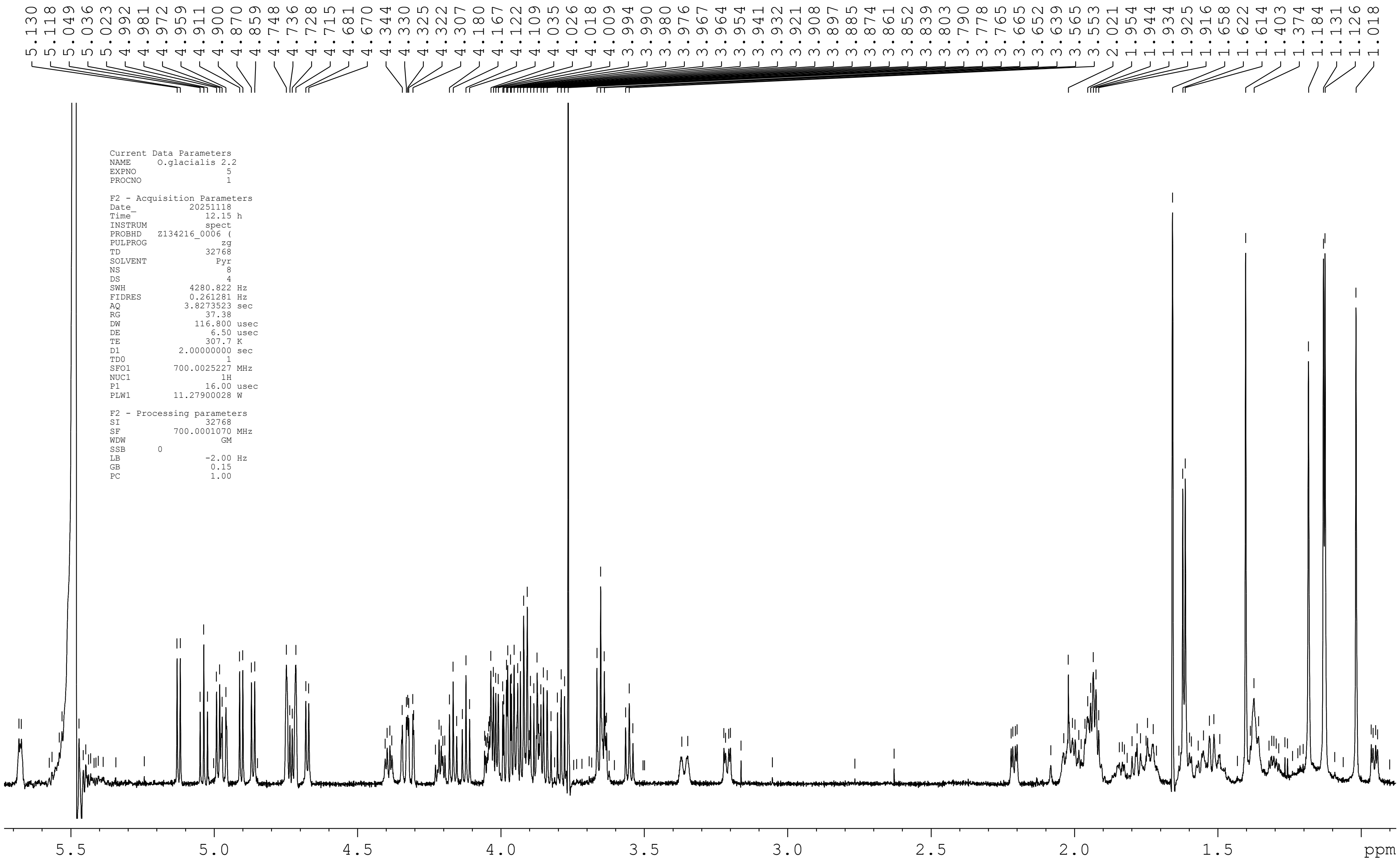

Figure S10. The ^{1}H NMR (700.13 MHz) spectrum of glacialisoside B (**2**) in C_5D_5N/D_2O (4/1)

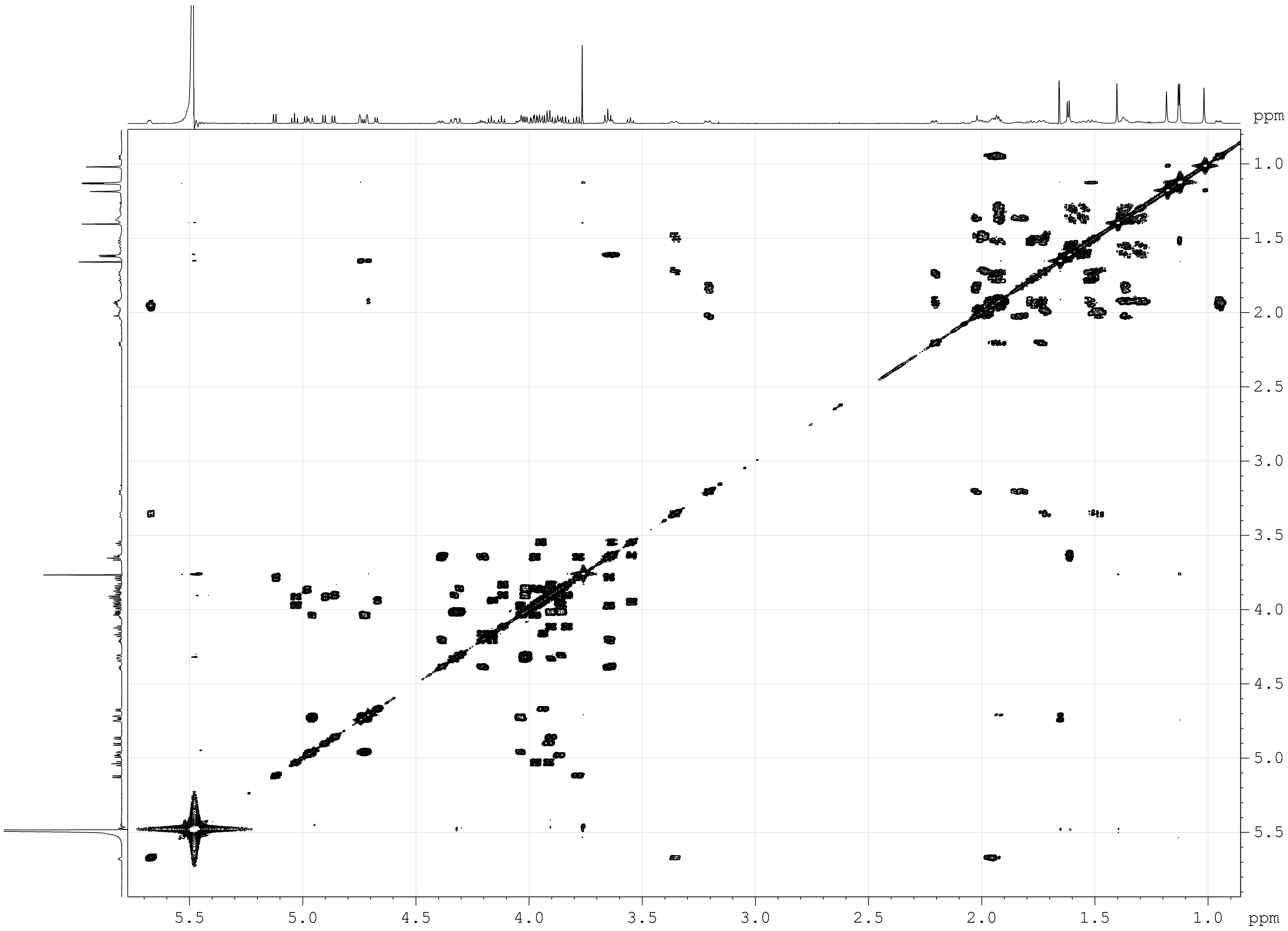

Figure S11. The COSY (700.13 MHz) spectrum of glacialisoside B (**2**) in C_5D_5N/D_2O (4/1)

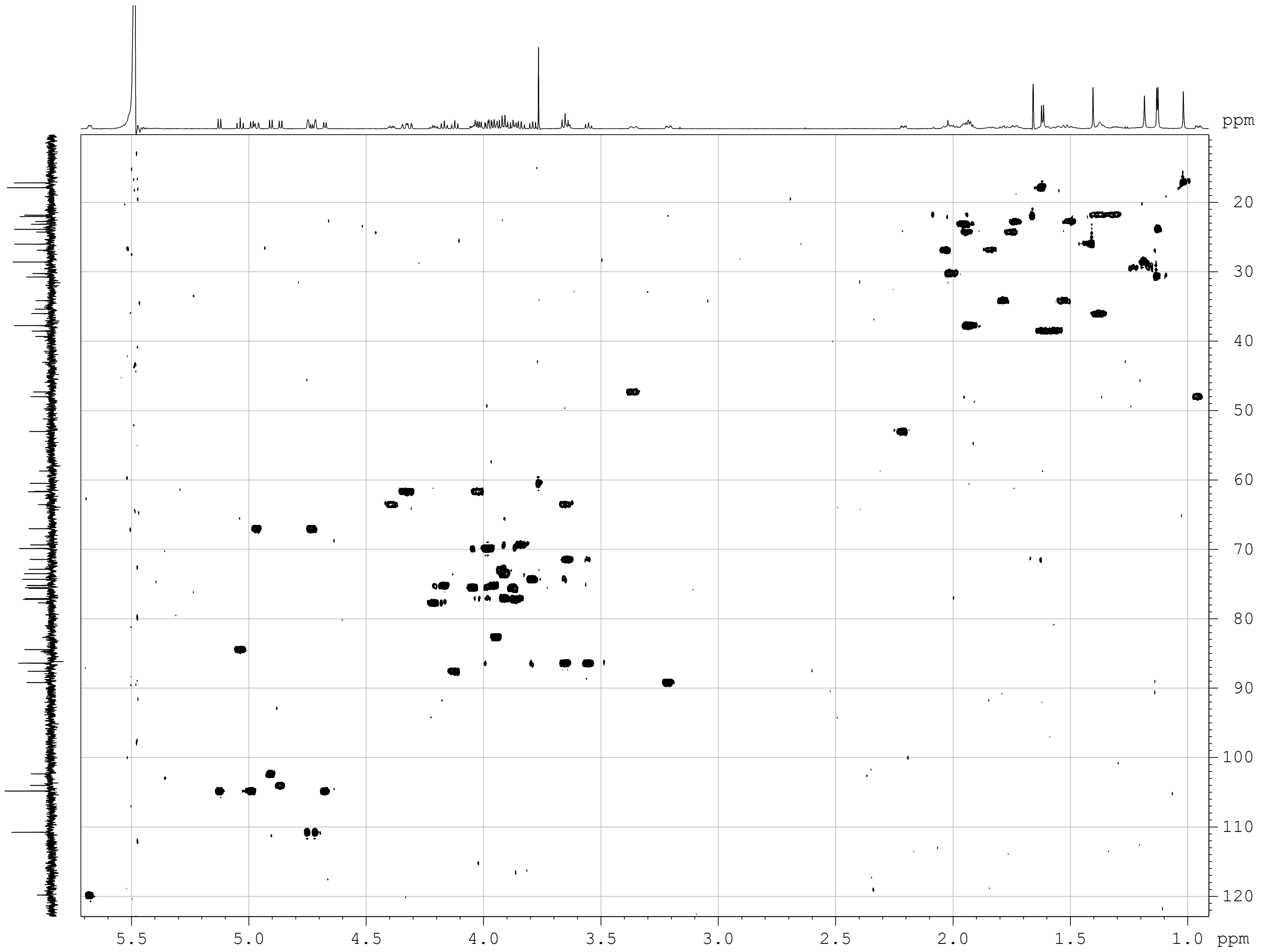

Figure S12. The HSQC (700.13 MHz) spectrum of glacialisoside B (**2**) in C_5D_5N/D_2O (4/1)

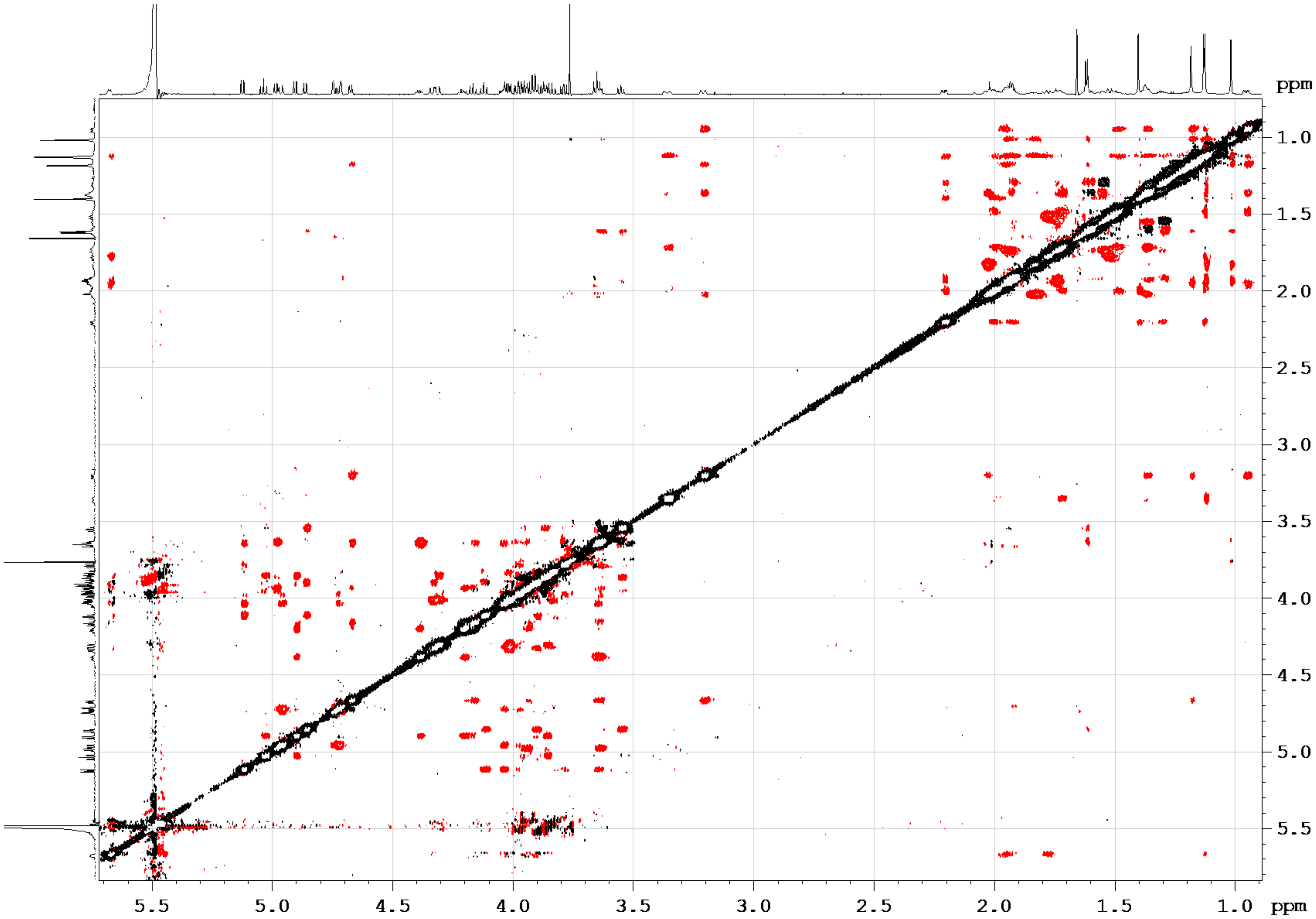

Figure S13. The ROESY (700.13 MHz) spectrum of glacialisoside B (**2**) in C_5D_5N/D_2O (4/1)

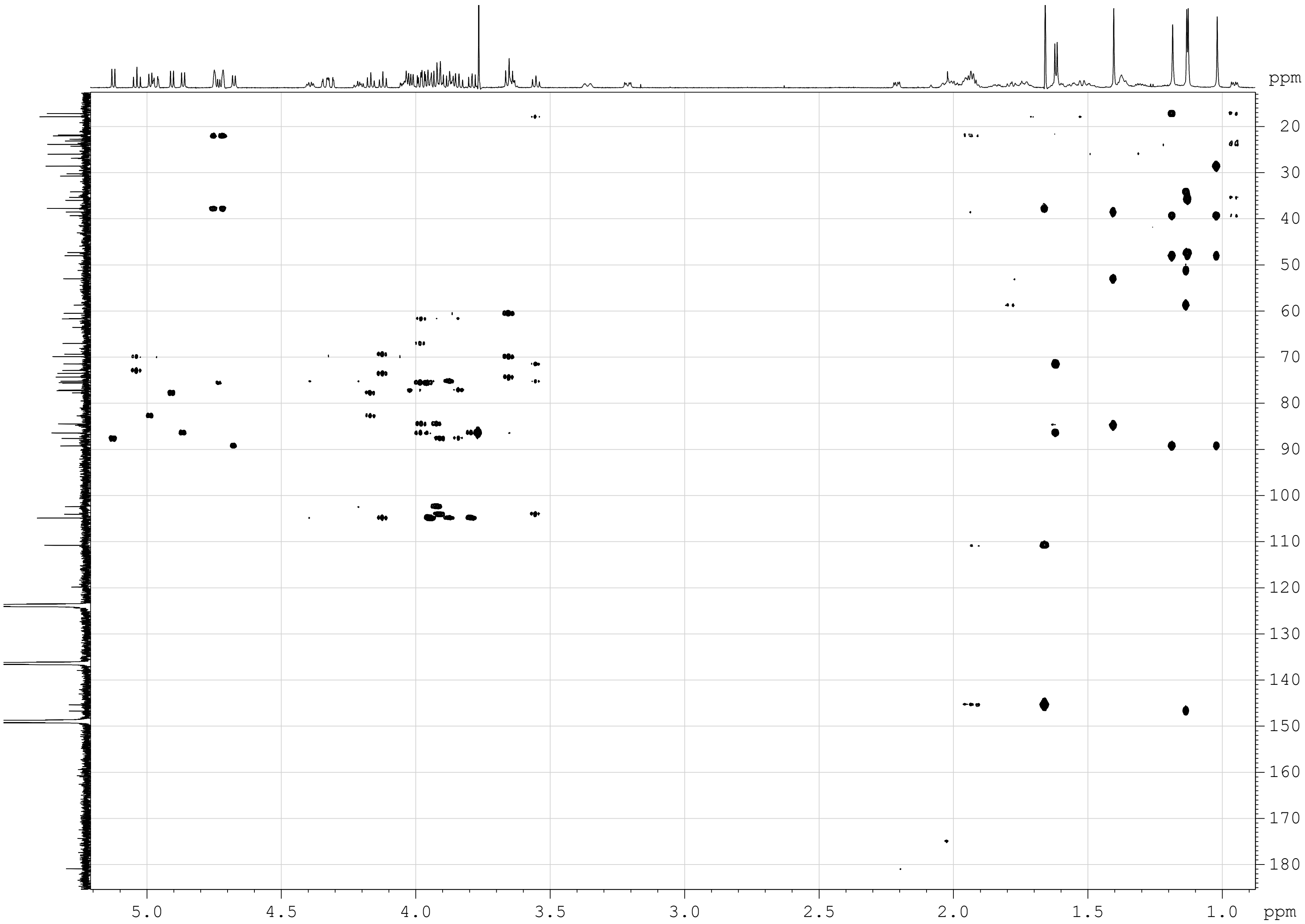

Figure S14. The HMBC (700.13 MHz) spectrum of glacialisoside B (**2**) in C_5D_5N/D_2O (4/1)

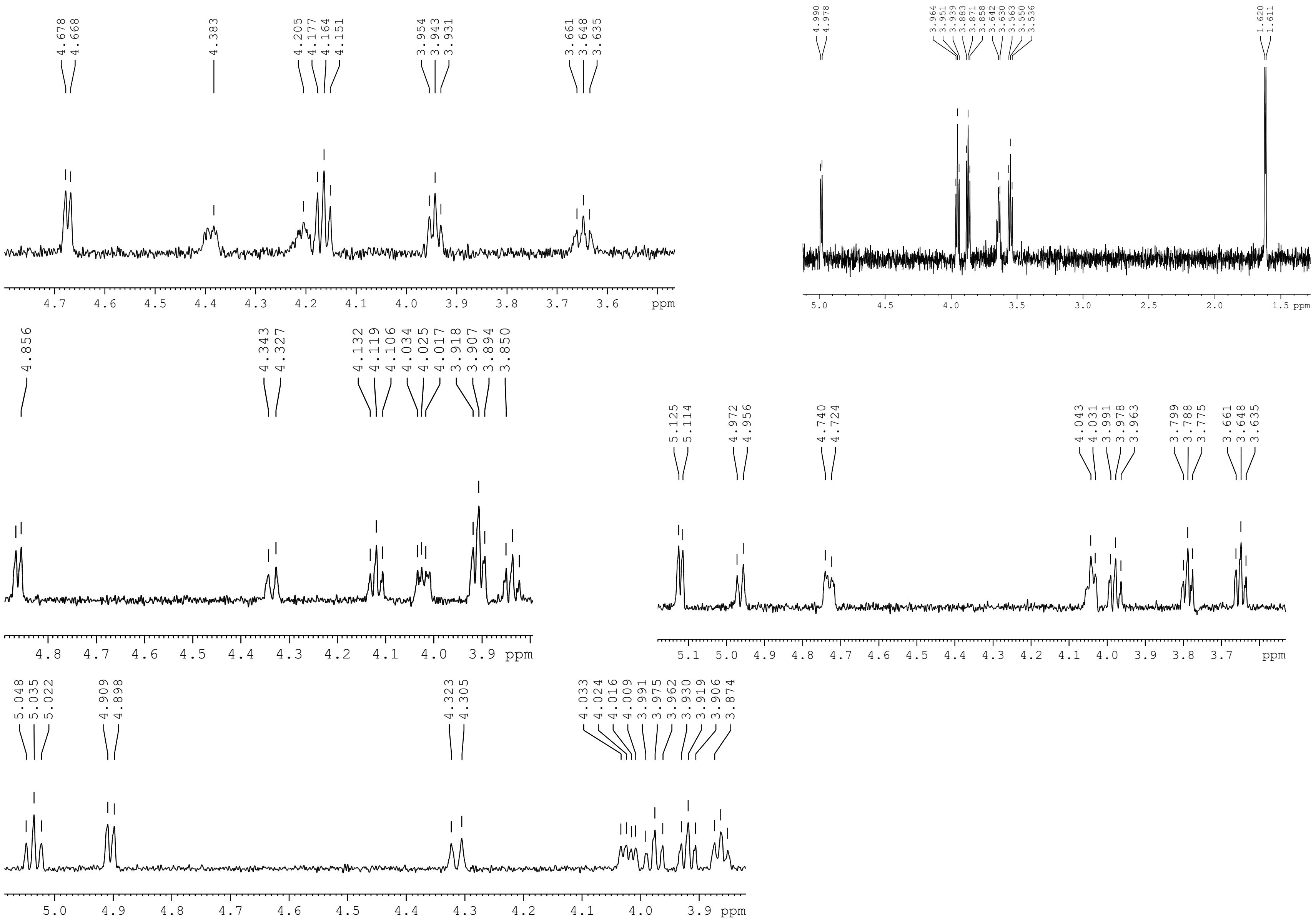

Figure S15. 1 D TOCSY (700.13 MHz) spectra of Xyl1, Qui2, Glc3, MeGlc4, Glc5 of glacialisoside B (**2**) in C_5D_5N/D_2O (4/1)

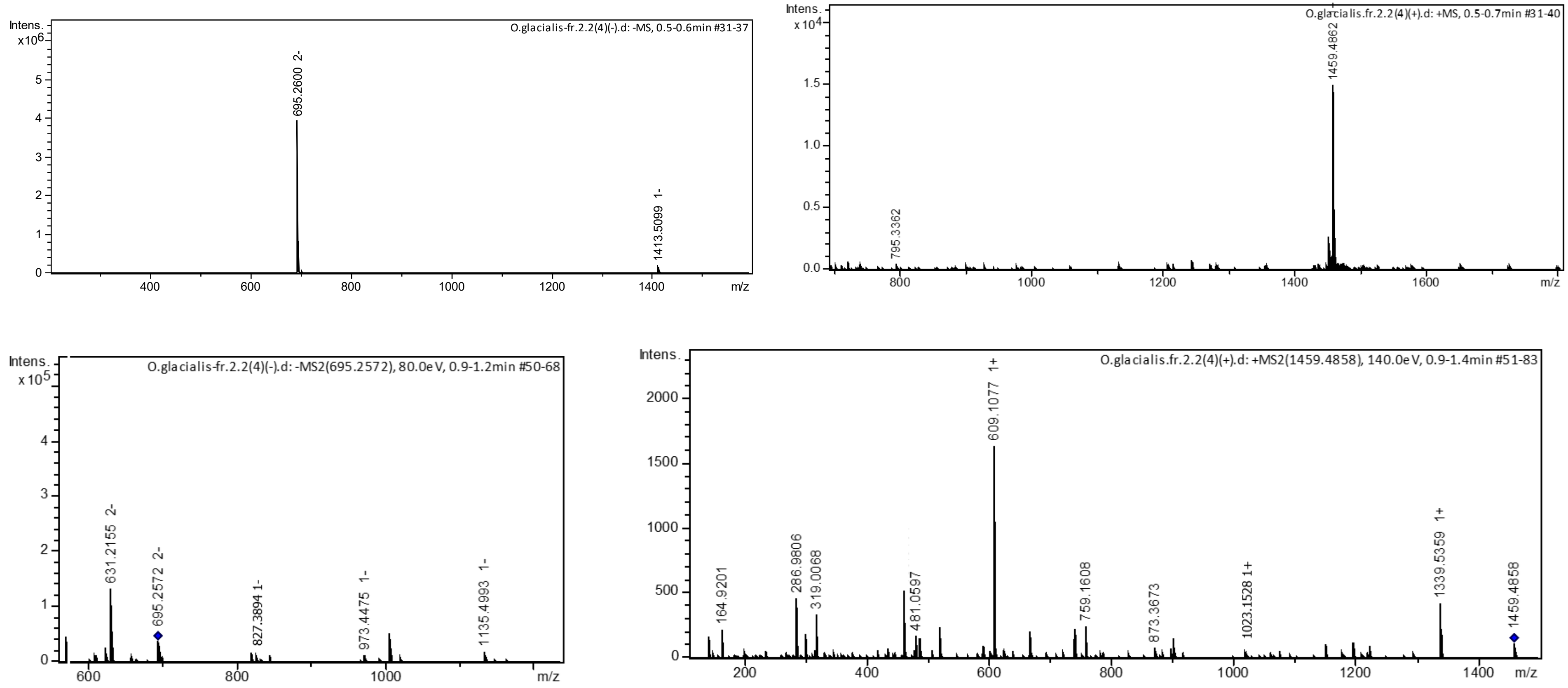

Figure S16. HR-ESI-MS and ESI-MS/MS spectra of glacialisoside B (**2**)

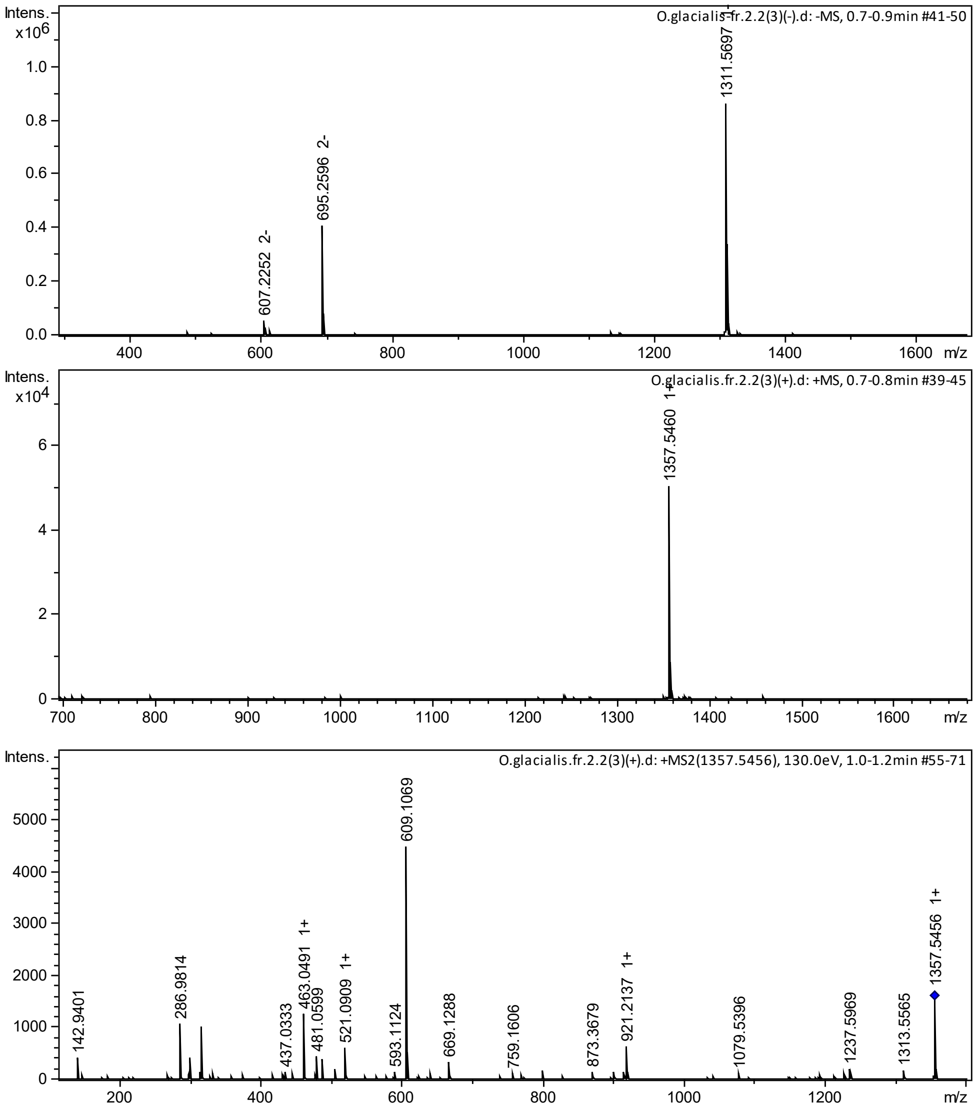

Figure S17. HR-ESI-MS and ESI-MS/MS spectra of **1** obtained as a result of solvolytic desulfation

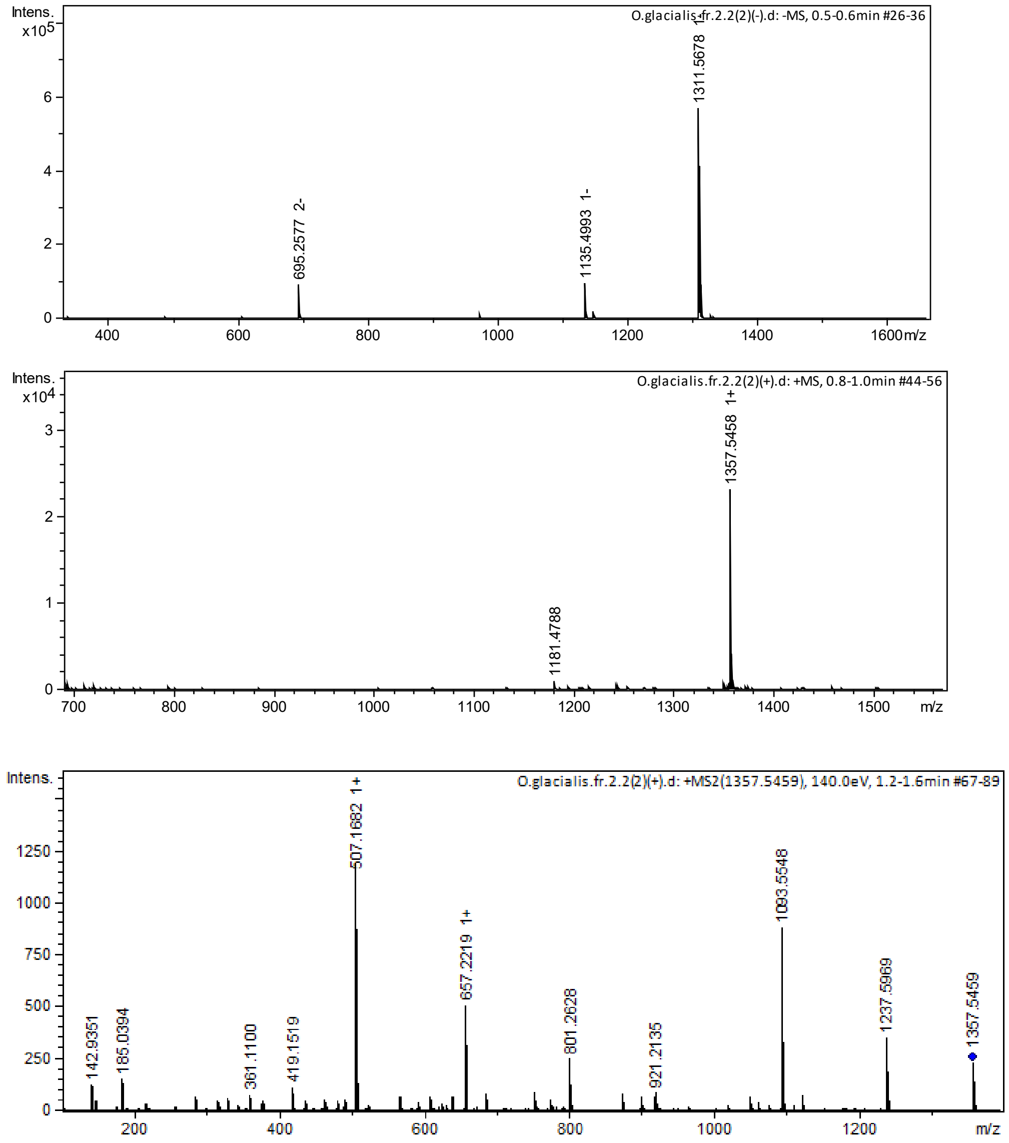

Figure S18. HR-ESI-MS and ESI-MS/MS spectra of DS-glacialisoside B (**3**)

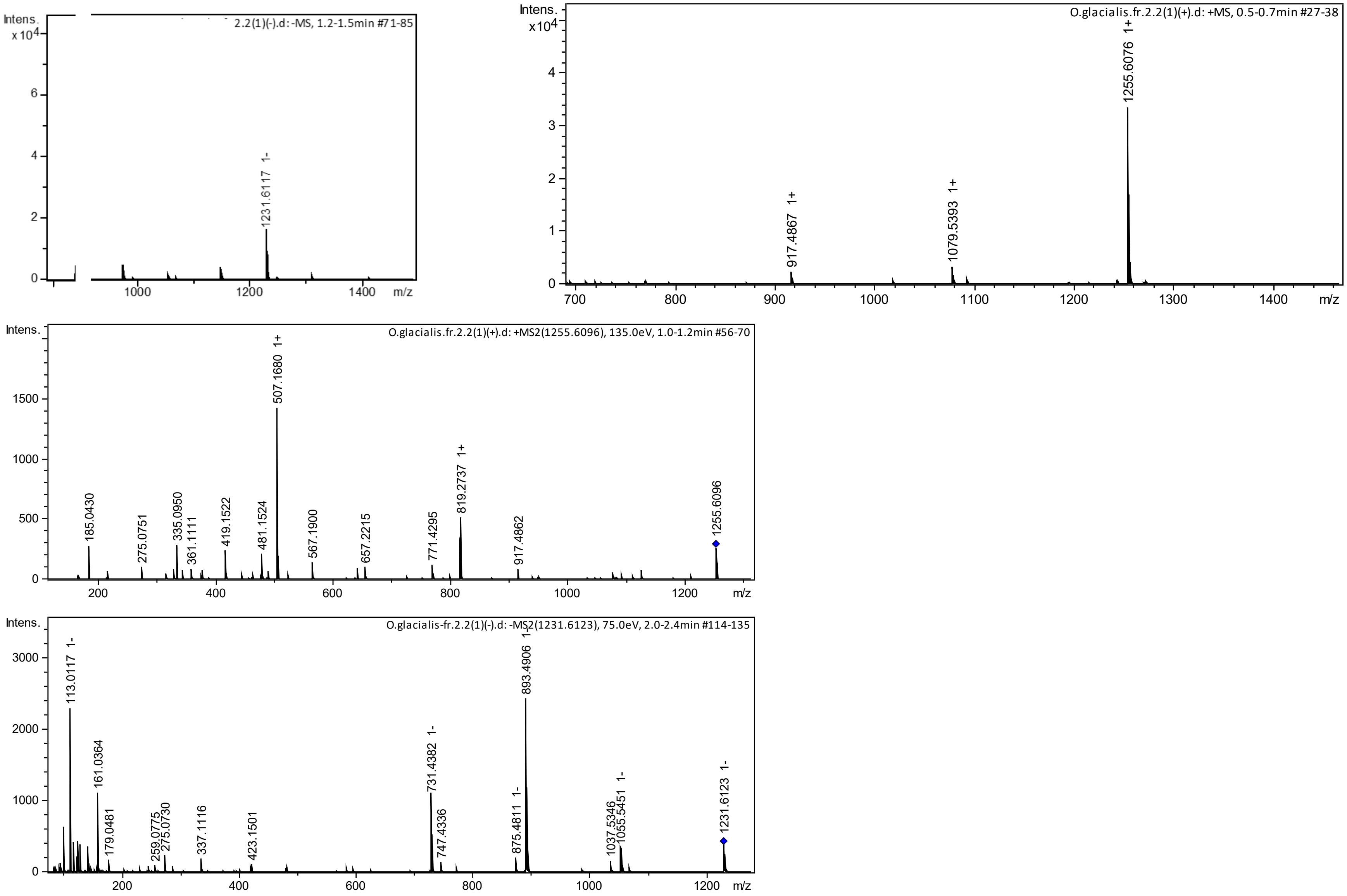

Figure S19. HR-ESI-MS and ESI-MS/MS spectra of di-DS-glacialisoside B (**4**)

Table S1. One- and two-dimensional NMR data of aglycone moiety of glacialisoside B (**2**)

Position	δC mult.[a]	δH mult. (*J* in Hz)[b]	HMBC	ROESY
1	36.0 CH_2	1.38 m		H-3, H-5, H-11, H-19
2	26.9 CH_2	2.03 m		
		1.84 m		H-19, H-30
3	89.2 CH	3.22 dd (11.6; 4.2)		H-1, H-5, H-31, H-1 Xyl1
4	39.3 C			
5	48.0 CH	0.95 dd (11.0; 4.3)		H-1, H-3, H-19, H-31
6	23.1 CH_2	1.95 m		
7	119.8 CH	5.68 m		H-15, H-32
8	146.7 C			
9	47.3 CH	3.36 brd (14.6)		H-19
10	35.4 C			
11	22.8 CH_2	1.73 m		H-1
		1.50 m		
12	30.2 CH_2	2.00 m		H-17, H-21
13	58.7 C			
14	51.2 C			
15	34.1 CH_2	1.78 m		H-7, H-32
		1.53 m		
16	24.3 CH_2	1.94 m		H-32
		1.75 m		
17	53.0 CH	2.21 dd (10.4; 3.7)	C: 13, 18, 21	H-12, H-21, H-23, H-32
18	180.9 C			
19	23.9 CH_3	1.12 s	C: 1, 9	H-1, H-2, H-6, H-9, H-30
20	84.7 C			
21	26.0 CH_3	1.40 s	C: 17, 20, 22	H-12, H-17
22	38.5 CH_2	1.62 m		H-24
		1.55 m		H-24
23	21.8 CH_2	1.37 m		
		1.30 m		H-17
24	37.7 CH_2	1.93 m		H-22, H-26
25	145.3 C			
26	110.7 CH_2	4.75 brs	C: 24, 27	
		4.72 brs	C: 24, 27	
27	22.0 CH_3	1.66 s	C: 24, 25, 26	
30	17.2 CH_3	1.02 s	C: 3, 4, 5, 31	H-2, H-6, H-19, H-31
31	28.6 CH_3	1.18 s	C: 3, 4, 5, 30	H-3, H-5, H-6, H-30, H-1 Xyl1
32	30.7 CH_3	1.13 s	C: 8, 13, 14, 15	H-7, H-15, H-16, H-17

[a] Recorded at 176.04 MHz in C_5D_5N/D_2O (4/1). [b] Recorded at 700.13 MHz in C_5D_5N/D_2O (4/1).
